# Conjugated Polymer Composite Nanoparticles Augmenting Photosynthesis‐Based Light‐Triggered Hydrogel Promotes Chronic Wound Healing

**DOI:** 10.1002/advs.202304048

**Published:** 2023-11-29

**Authors:** Qiong Yuan, Jia Yin, Ling Li, Benkai Bao, Xinyi Zhang, Meiqi Li, Yanli Tang

**Affiliations:** ^1^ Key Laboratory of Applied Surface and Colloid Chemistry Ministry of Education Key Laboratory of Analytical Chemistry for Life Science of Shaanxi Province School of Chemistry and Chemical Engineering Shaanxi Normal University Xi'an 710119 P. R. China

**Keywords:** conjugated polymer nanoparticles, hypoxia, light‐triggered hydrogel, photosynthesis, wound healing

## Abstract

Diabetic chronic wounds are characterized by local hypoxia, impaired angiogenesis, and bacterial infection. In situ, self‐supply of dissolved oxygen combined with the elimination of bacteria is urgent and challenging for chronic nonhealing wound treatment. Herein, an oxygen‐generating system named HA‐L‐NB/PFE@cp involving biological photosynthetic chloroplasts (cp)/conjugated polymer composite nanoparticles (PFE‐1‐NPs@cp) and light‐triggered hyaluronic acid‐based (HA‐L‐NB) hydrogel for promoting diabetic wound healing is introduced. Briefly, conjugated polymer nanoparticles (PFE‐1‐NPs) possess unique light harvesting ability, which accelerates the electron transport rates in photosystem II (PS II) by energy transfer, elevating photosynthesis beyond natural chloroplasts. The enhanced release of oxygen can greatly relieve hypoxia, promote cell migration, and favor antibacterial photodynamic therapy. Additionally, the injectable hydrogel precursors are employed as a carrier to deliver PFE‐1‐NPs@cp into the wound. Under light irradiation, they quickly form a gel by S‐nitrosylation coupling reaction and in situ anchor on tissues through amine‐aldehyde condensation. Both in vitro and in vivo assays demonstrate that the oxygen‐generating system can simultaneously relieve wound hypoxia, eliminate bacteria, and promote cell migration, leading to the acceleration of wound healing. This study provides a facile approach to develop an enhanced oxygen self‐sufficient system for promoting hypoxic tissue, especially diabetic wound healing.

## Graphical abstract

A novel oxygen generating system HA‐L‐NB/PFE@cp by combining conjugated polymers/chloroplast composite nanoparticles with photo‐triggered hydrogels is constructed. The enhanced photosynthesis of composite nanoparticles effectively relieves hypoxia, promotes cell migration, and favors photodynamic antibacterial. Hydrogels can quickly anchor on tissues. Both in vitro and in vivo assays demonstrate excellent chronic wounds healing effect.

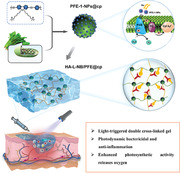

## 1 Introduction

Diabetes mellitus (DM), as a chronic metabolic disease, is a leading cause of nonhealing wounds.^[^ ^1^ ^]^ Chronic nonhealing wounds are mainly caused by the following reasons: 1) The insufficient blood supply of the local wound.^[^ ^2^ ^]^ 2) Neurotrophic deficiency.^[^ ^3^ ^]^ Vascular lesions usually occur around the wound, further aggravating the local blood supply deficiency, because of the long course of DM. Peripheral vascular circulation disorder, infection, and local inflammatory cells will consume a large amount of oxygen for respiratory burst in the ischemic state, resulting in local hypoxia of the wound, which further leads to delayed healing.^[^ ^4^, ^5^, ^6^, ^7^ ^]^ Thus, enhancing wound tissue oxygenation is a key to promote chronic wound healing in DM. Hyperbaric oxygen therapy (HBOT) and topical gaseous oxygen (TGO) therapy can be used to relieve wound hypoxia, reduce edema, and down‐regulate inflammatory factors.^[^ ^8^, ^9^, ^10^, ^11^, ^12^ ^]^ Nevertheless, during treatment with HBOT, oxygen levels are only elevated temporarily. Furthermore, HBOT may result in risks of tissue hyperoxia.^[^ ^13^ ^]^ TGO treatment is limited by the permeability of external gases to the tissue. In addition, the hyperglycemic environment in the wound of diabetic patients provides conditions for bacterial colonization and growth, which can easily lead to multiple mixed bacterial infections.^[^ ^14^, ^15^, ^16^, ^17^ ^]^ Therefore, the in situ self‐supply of dissolved oxygen at the wound site combined with the elimination of bacteria is urgent and challenging for chronic nonhealing wound treatment.

Photosynthesis plays a vital role in synthesizing carbohydrates and delivering oxygen with the help of sunlight. The main processes of the photosynthetic light reaction are carried out by the protein complexes photosystem I (PS I) and photosystem II (PS II) in the chloroplast, which ultimately produce oxygen and ATP by splitting water.^[^ ^18^, ^19^, ^20^ ^]^ However, the absorbed light by chloroplasts is limited to the visible spectrum during the photosynthesis process. In order to increase the solar energy conversion efficiency of chloroplasts, various light‐harvesting nanomaterials, such as single‐walled carbon nanotubes and double‐emitting carbon dots, have been employed to convert poorly absorbed UV light into highly absorbed visible light.^[^ ^21^, ^22^, ^23^, ^24^, ^25^ ^]^ Thus, the development of hybrid photosynthetic system involving chloroplasts is highly desirable for enhancing solar energy utilization efficiency and improving the photosynthesis efficacy.

In recent years, conjugated polymers (CPs) have obtained much attention in organic light‐emitting diodes, organic solar cells, biosensors, bioimaging, et al., due to high molar absorption coefficient, high fluorescence quantum yield, mechanical flexibility, and semiconductors properties.^[^ ^26^, ^27^, ^28^, ^29^, ^30^ ^]^ Also, CPs can generate active oxygen or heat energy under light excitation and have been extensively applied in the treatment of tumors and pathogenic bacteria infections.^[^ ^31^, ^32^ ^]^ In addition, CPs can quickly transfer excitons to nearby low‐energy acceptors just like the energy transfer (ET) between antenna pigments in photosynthesis.^[^ ^33^, ^34^ ^]^ It means that CPs can be used as artificial antennas to compensate for the lack of natural antenna pigments to improve the efficiency of photosynthesis and oxygen production. For example, Wang et al.^[^ ^35^ ^]^ have designed and constructed a hybrid biosystem by electrostatically recombining conjugated polymer with a cyanobacterium to enhance photosynthesis and regulate the exogenous redox state of a protein. Qi et al.^[^ ^23^ ^]^ reported an in situ polymerization strategy to modify cell surface with CPs for augmenting the ATP synthesis of *Chlorella pyrenoidosa*. Inspired by the aforementioned findings, exploring engineering CPs hybrid materials for augmenting photosynthesis, improving oxygen production, and enhancing sterilization is an ideal strategy for chronic wound healing.

**Figure 1:**
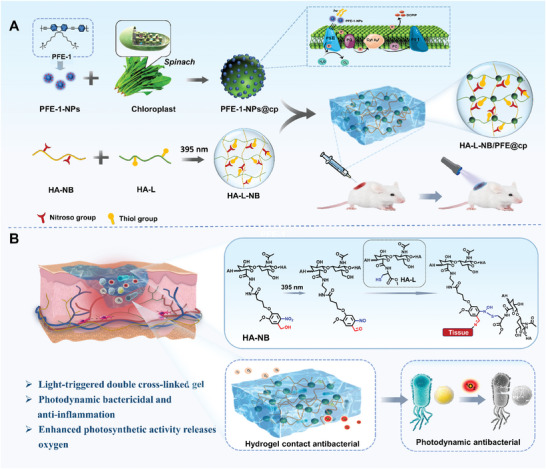
Illustration of the gelation mechanism, enhancement of photosynthesis of chloroplasts, and chronic wound healing utilizing HA‐L‐NB/PFE@cp hydrogel. A) Preparation process of HA‐L‐NB/PFE@cp hydrogel and oxygen production mechanism. B) The scheme of HA‐L‐NB/PFE@cp hydrogel for promoting hypoxic wound healing by realizing in situ gelation, photodynamic bactericidal, relieving wound hypoxia, and promote cell migration.

Herein, we presented a novel oxygen‐generating system HA‐L‐NB/PFE@cp based on biological photosynthetic chloroplasts/conjugated polymer composite nanoparticles (PFE‐1‐NPs@cp) and light‐triggered HA‐L‐NB hydrogel. As shown in **Figure** **1**, PFE‐1‐NPs@cp can be delivered to the wound site by HA‐L‐NB hydrogel, which has light‐triggered and rapid gelation characteristics. The o‐nitrobenzyl carbinol labeled on the hyaluronic acid (HA) chain is converted into o‐nitrosobenzaldehyde by photochemical reaction upon 395 nm illumination. The generated nitroso group is coupled with the thiol group on the other HA branch through a rapid S‐nitrosylation (SNO) coupling reaction, resulting in the gelation of the hydrogel. Simultaneously, photogenerated aldehyde groups are anchored to the amino groups on the tissue, achieving in situ adhesion. In PFE‐1‐NPs@cp complex, the absorption spectra of chloroplasts and the emission spectra of PFE‐1‐NPs have a good overlap, which will trigger the ET and enhance the light utilization, resulting in the higher photosynthetic efficiency. In addition, PFE‐1‐NPs@cp can generate a large amount of reactive oxygen species (ROS) under light irradiation to realize photodynamic sterilization, further preventing wound infection. In vivo assays showed that this system can realize in situ gelation, relieve wound hypoxia, eliminate bacteria, and finally accelerate wound healing. This study also highlights the role of artificial antennas composed of photosynthetic organisms and organic interfaces as oxygen‐generating agents, which provides new insights into the treatment of chronic wounds and other hypoxia‐related disorders.

## 2 Results and Discussion

### 2.1 Preparation and Characterization of PFE‐1‐NPs@cp

**Figure 2:**
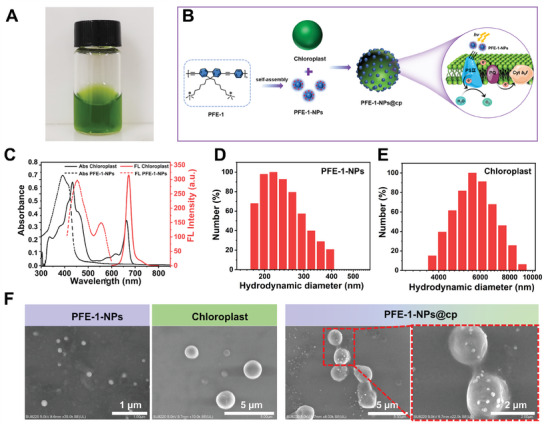
Synthesis and characterization of PFE‐1‐NPs@cp composite nanoparticles. A) Photographs of intact chloroplast suspensions extracted from fresh spinach leaves. B) Schematic illustration of PFE‐1‐NPs@cp and the mechanism of oxygen release. C) Absorption and emission spectra of PFE‐1‐NPs and chloroplasts. Size distributions of D) PFE‐1‐NPs and E) chloroplasts. F) SEM images of PFE‐1‐NPs, chloroplasts, and PFE‐1‐NPs@cp.

To prepare PFE‐1‐NPs@cp, cationic poly(fluorene‐co‐phenylene ethynylene) derivatives (PFE‐1) were synthesized according to the literature (Figure S1, Supporting Information), and its nanoparticles PFE‐1‐NPs were prepared by the nanoprecipitation method.^[^ ^36^ ^]^ The photosynthetic units, chloroplasts, were extracted from fresh spinach leaves by the double‐gradient method (**Figure** **2**A), which contain negatively charged outer membranes due to the presence of hydroxyl, carboxyl, and phosphate groups in phospholipids. Then, as shown in Figure 2B, PFE‐1‐NPs@cp was constructed by electrostatically recombining photoactive cationic PFE‐1‐NPs with chloroplasts to enhance photosynthetic efficiency. The photosynthesis in chloroplasts relies on critical protein complex photosystem II (PS II) to capture light to split water into protons, oxygen, and electrons.^[^ ^37^, ^38^ ^]^ PFE‐1‐NPs have excellent light capture ability, especially for ultraviolet light. Under light irradiation, PFE‐1‐NPs on the surface of chloroplasts emit blue light that can be absorbed by chloroplasts, Thus, more light can be captured by PS II to oxidize water and generate more electrons in the electron transport chain, ultimately increasing photosynthetic activity and oxygen production. As shown in Figure 2C, PFE‐1‐NPs exhibited strong ultraviolet light absorption at the maximum absorption wavelength of 393 nm, while chloroplasts showed broad absorption in blue and red fluorescence regions at 350–500 and 630–680 nm, respectively. Moreover, the emission scope of PFE‐1‐NPs was located at 400–524 nm, which had excellent overlap with chloroplasts absorption, enabling great ET between them.^[^ ^34^ ^]^ In addition, the hydration particle sizes of PFE‐1‐NPs and chloroplasts were characterized by dynamic light scattering (DLS) (Figure 2D,E). The main size of PFE‐1‐NPs and chloroplasts was ≈218 nm and 5 µm, respectively. Then, scanning electron microscopy (SEM) images were obtained to characterize the prepared PFE‐1‐NPs@cp (Figure 2F). After incubation with PFE‐1‐NPs, some attached particles were found on the surface of chloroplasts, which proved the successful coating of PFE‐1‐NPs on chloroplasts.

### 2.2 Enhancement of Photosynthetic Activity of Chloroplasts

**Figure 3:**
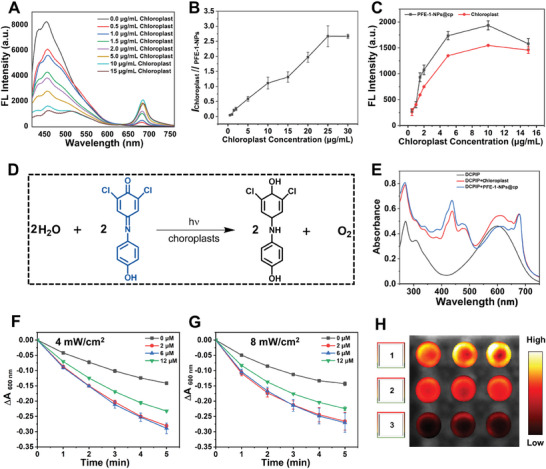
ET effect and photosynthetic activity evaluation of PFE‐1‐NPs@cp. A) Fluorescence spectra of PFE‐1‐NPs@cp containing different concentrations of chloroplasts. B) Fluorescence ratio of PFE‐1‐NPs@cp at different concentrations of chloroplasts. C) Fluorescence intensity of PFE‐1‐NPs@cp and chloroplast suspension. D) The reduction of DCPIP dye by chloroplasts (Hill reaction). E) Absorption spectra of DCPIP after incubation with chloroplasts and PFE‐1‐NPs@cp, respectively. The absorption changes of DCPIP with different concentrations of PFE‐1‐NPs under light irradiation at 4 mW cm^−2^ F) and 8 mW cm^−2^ G). (PFE‐1‐NPs) = 0, 2, 6, 12 µm. H) Detection of oxygen in D‐PBS solution under different treatments (1, control, 2, chloroplasts, 3, PFE‐1‐NPs@cp + Light) by ((Ru(dpp)_3_))Cl_2_ probe. The results represent mean ± standard deviation (*n* = 3).

In PFE‐1‐NPs@cp, PFE‐1‐NPs and chloroplasts may serve as a donor‐acceptor pair, enabling efficient ET from PFE‐1‐NPs to chloroplasts. To confirm this hypothesis, the fluorescence spectra of PFE‐1‐NPs@cp at different chloroplast concentrations were measured (**Figure** **3**A). The fluorescence intensity at 682 nm (λ_max_ of chloroplasts) enhanced and at 452 nm (λ_max_ of PFE‐1‐NPs) decreased with the increasing of chloroplast concentration, bringing an increase in the fluorescence intensity ratio (*I*_chloroplast_ */I*_PFE‐1‐NPs_) (Figure 3B). Furthermore, Figure 3C compared the fluorescence intensity of PFE‐1‐NPs@cp with that of chloroplasts alone, which revealed that the intensity at 682 nm significantly increased after the formation of PFE‐1‐NPs@cp, indicating that a valuable ET from PFE‐1‐NPs to chloroplasts occurred.

Moreover, the photosynthetic activity of PFE‐1‐NPs@cp was investigated by measuring the reduction rate of the dye 2,6‐dichlorophenolindophenol (DCPIP). DCPIP is an artificial electron acceptor that can capture electrons transported from PS II to PS I in the photoreaction of chloroplasts (Figure 3D). As shown in Figure 3E, the absorption maximum of DCPIP was at 600 nm where chloroplasts and PFE‐1‐NPs@cp had no obvious absorption. To screen for the optimal photosynthetic activity of PFE‐1‐NPs@cp, we investigated ΔA_600 nm_ in the presence of PFE‐1‐NPs@cp containing different concentrations of PFE‐1‐NPs and 5 µg mL^−1^ chloroplasts under different light intensities (4 and 8 mW cm^−2^). The ΔA_600 nm_ in the presence of chloroplasts gradually decreased with increasing irradiation time, which implied that the photosynthesis of chloroplasts themselves leads to DCPIP reduction (Figure 3F,G). In contrast, in the presence of PFE‐1‐NPs@cp, the reduction rate of DCPIP was obviously accelerated due to PFE‐1‐NPs with good light‐harvesting ability strongly absorbing ultraviolet light and transferring to chloroplasts, thereby effectively enhancing the photosynthetic activity of chloroplasts.^[^ ^39^ ^]^

Additionally, the oxygen‐deficient probe tris (4,7‐diphenyl‐1,10‐phenanthroline) ruthenium (II) dichloride ((Ru(dpp)_3_)Cl_2_) was used to detect oxygen generation of PFE‐1‐NPs@cp (Figure 3H). The fluorescence intensity of the probe is dependent on the degree of hypoxia.^[^ ^40^, ^41^ ^]^ It was obviously observed that the fluorescence of PFE‐1‐NPs@cp + Light group decreased more significantly under light illumination compared to that of chloroplasts, indicating that more oxygen was generated from PFE‐1‐NPs@cp than from chloroplasts themselves. These results demonstrated that PFE‐1‐NPs can enhance the photosynthetic activity of chloroplasts and promote the generation of oxygen, which was attributed to the strong absorption ability in the UV range and the effective ET effect from PFE‐1‐NPs to chloroplasts.

### 2.3 PFE‐1‐NPs@cp Improved Oxygen Production and Promoted Cells Migration

**Figure 4:**
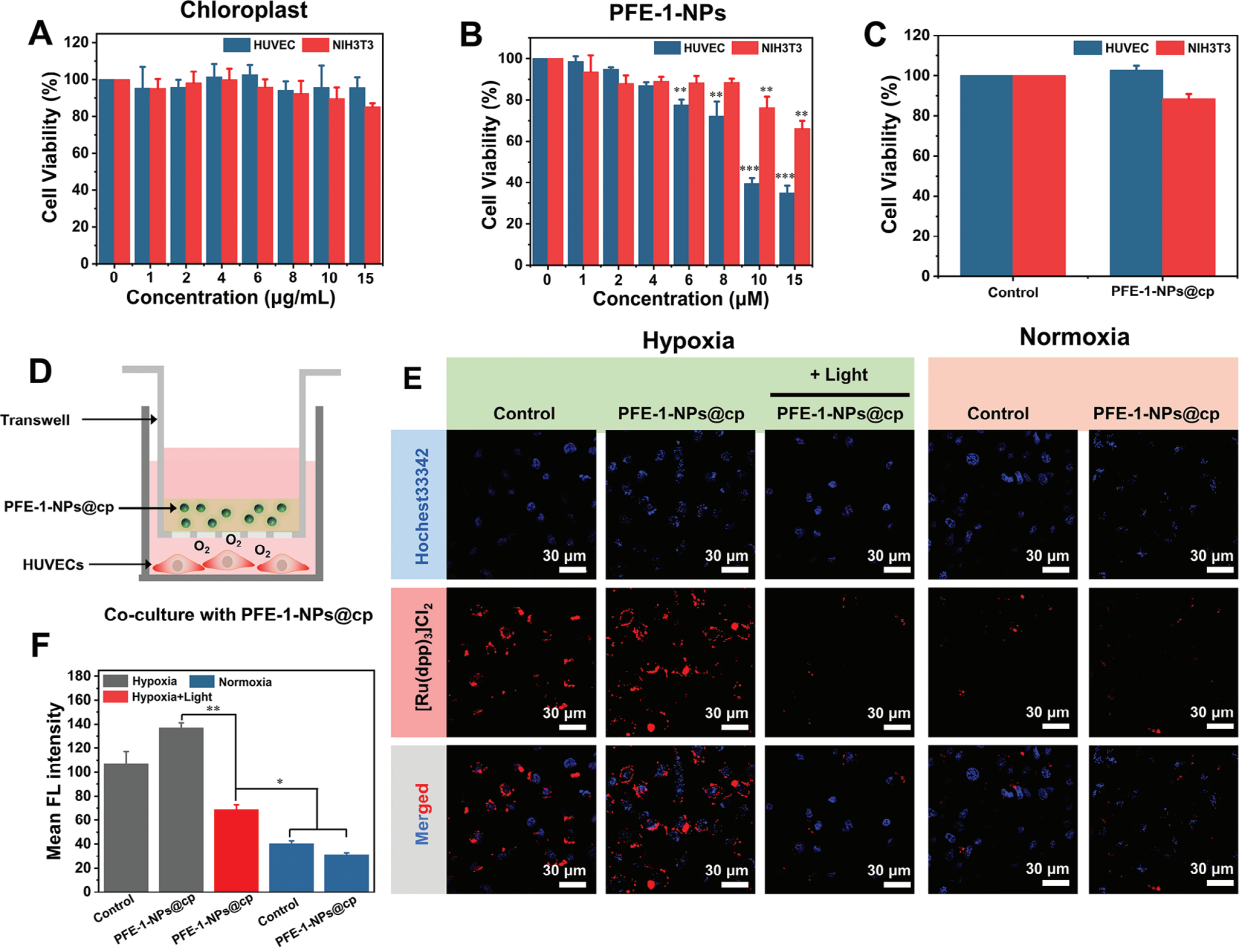
Oxygen production by PFE‐1‐NPs@cp. A,B) Cell viability treated with PFE‐1‐NPs and chloroplasts. C) Cell viability treated with PFE‐1‐NPs@cp. (PFE‐1‐NPs) = 6 µm, (chloroplast) = 5 µg mL^−1^. D) Schematic illustration of the design scheme with PFE‐1‐NPs@cp in vitro. E) CLSM images of intracellular hypoxia levels at hypoxic and normoxic conditions with different treatments. Scale bar = 30 µm, blue, fluorescence of Hoechst 33 342; red, fluorescence of hypoxia probe; overlay images. F) Mean FL intensity calculated from E). The results represent mean ± standard deviation (*n* = 3). ^*^ *p* < 0.05, ^**^ *p* < 0.01, ^***^ *p* < 0.001 (Student's *t*‐test).

First, the cytotoxicity of chloroplast and PFE‐1‐NPs were evaluated by two normal cells, human umbilical vein endothelial cells (HUVECs) and fibroblasts NIH3T3 cells. As shown in **Figure** **4**A, HUVECs and NIH3T3 cells exhibited a high cell survival rate after treatment with various concentrations of chloroplasts, indicating the great biocompatibility of chloroplasts to normal cells. When the concentration of PFE‐1‐NPs was 6 µm, the cell survival rate remained about 80% in Figure 4B, which indicated that PFE‐1‐NPs had low cytotoxicity and provided a prerequisite for their application in the biological field. Also, the high cell viability of both HUVECs and NIH3T3 cells showed that PFE‐1‐NPs@cp had great biocompatibility (Figure 4C). In addition, the oxygen‐producing cytotoxicity of PFE‐1‐NPs@cp was studied by the device in Figure 4D. The cytotoxicity test found that under 395 nm light illumination (4 mW cm^−2^, 30 min), the survival rates of two kinds of cells (HUVECs and NIH3T3 cells) in both chloroplast group and PFE‐1‐NPs@cp group exceeded 95%, and the morphology of cells was unchanged compared to the control, indicating that the oxygen produced during the experiments was not cytotoxic (Figure S3, Supporting Information).

Diabetic wound healing is accompanied by peripheral vascular circulation disorders and infection. Locally generated inflammatory cells consume large amounts of oxygen for respiratory bursts, resulting in an increased demand for oxygen. Thus, the local wound is in a state of hypoxia. In order to simulate the high glucose and hypoxia environment, the most suitable glucose concentration was screened by MTT assay. In hypoxic conditions, the cell survival rate decreased with the increase in glucose concentration. The cell viability of HUVECs incubated under hypoxic conditions for 24 h was less than 80% at 30 mm glucose (Figure S4, Supporting Information). Therefore, 30 mm of glucose concentration and hypoxia conditions were used to simulate the diabetic wound environment.

To investigate the effect of PFE‐1‐NPs@cp on intracellular hypoxia levels after light irradiation, HUVECs cells were seeded in the lower chamber of light‐transmitting transwell, and PFE‐1‐NPs@cp were added to the upper chamber at hypoxic condition (Figure 4D). The intracellular oxygen produced from PFE‐1‐NPs@cp was detected by Ru(dpp)_3_Cl_2_ probe. As shown in Figure 4E, the control and PFE‐1‐NPs@cp groups showed bright red fluorescence at hypoxic conditions. However, under 395 nm light illumination, even at the same condition, PFE‐1‐NPs@cp groups showed very weak red fluorescence, which was similar to that of unilluminated cells at normoxic conditions. The quantitative FL intensity of PFE‐1‐NPs@cp at hypoxia + Light group significantly decreased (Figure 4F). These results demonstrated that PFE‐1‐NPs@cp + Light had excellent oxygen production capacity under hypoxia.

**Figure 5:**
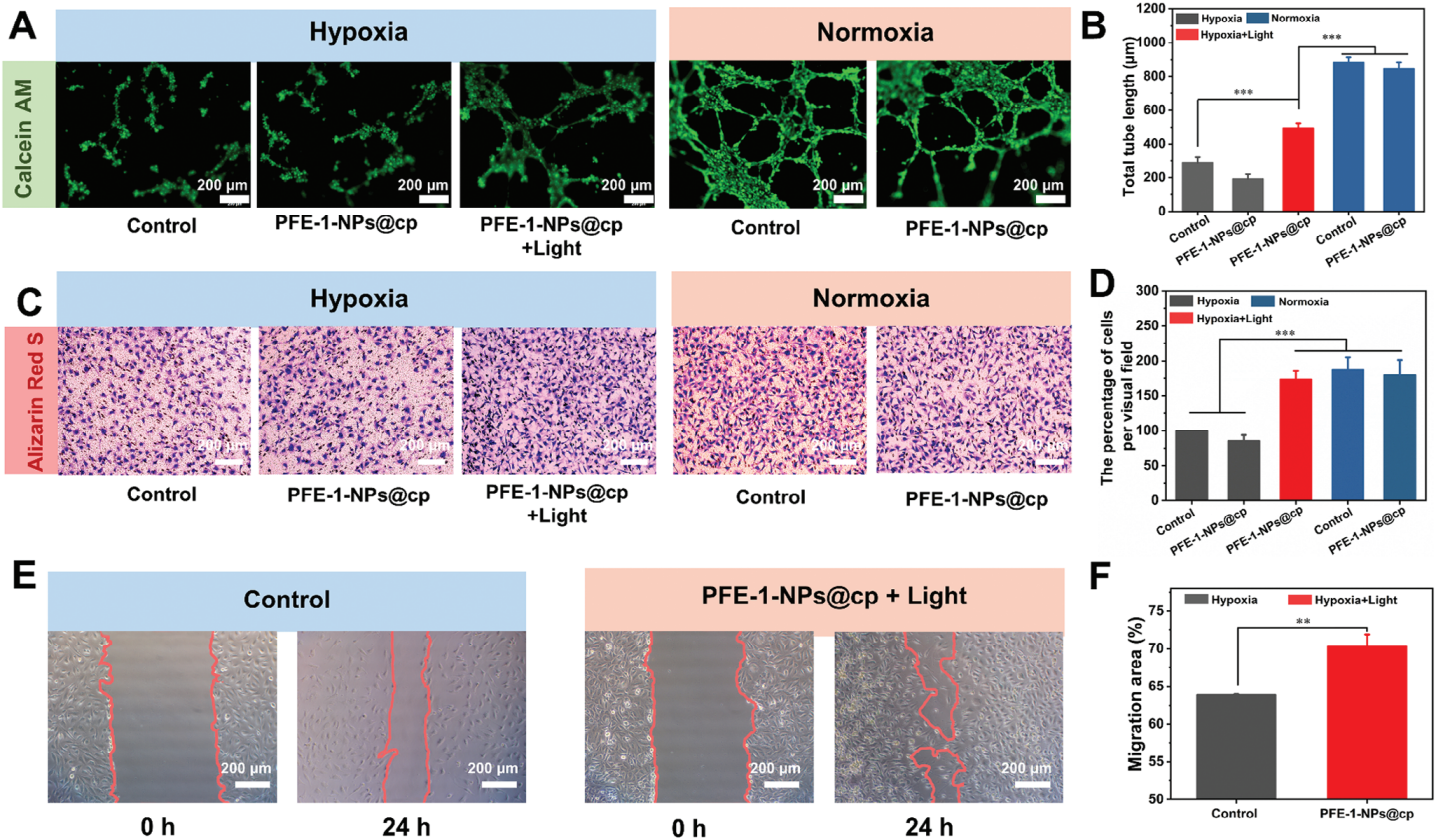
In vitro activity evaluation of PFE‐1‐NPs@cp. A) Representative images of HUVECs’ tube formation at hypoxic and normoxic conditions with different treatments. B) Quantification of HUVECs’ tube formation. C) Representative images of the transwell migration assay with different treatments. D) Quantification of transwell migration assay. E) Representative images of the scratch assay with different treatments. F) Quantitative measurement of the scratched area. The results represent mean ± standard deviation (*n* = 3). Scale bar = 200 µm. ^*^ *p* < 0.05, ^**^ *p* < 0.01, ^***^ *p* < 0.001 (Student's *t*‐test).

HUVECs were chosen to perform tube formation experiments. As shown in **Figure** **5**A, compared with the normoxic groups (control, PFE‐1‐NPs@cp), the hypoxic groups had minimal mature branch points and capillary‐like structures, indicating that the hypoxia inhibited the angiogenic potential of the cells. Interestingly, the branching point number and vessel formation were increased significantly in PFE‐1‐NPs@cp groups under light irradiation even at hypoxic conditions (Figure 5B) (*p* < 0.001). High glucose and hypoxic microenvironment will lead to the decrease of cell migration ability in wounds. Therefore, the migration ability of HUVECs with different treatments was evaluated by the transwell method and scratch assay. As shown in Figure 5C, under hypoxic conditions, both the control group and PFE‐1‐NPs@cp group exhibited reduced migratory ability. In the PFE‐1‐NPs@cp group, the average percentage of migrated cells was 85.6%± 8.2%, as observed under the microscope. After light exposure, the migration of cells in the PFE‐1‐NPs@cp group increased (171%± 11.9%) through the polycarbonate filter, which showed no significant difference with the control group (187.2%± 17.9%) and the PFE‐1‐NPs@cp group (180%± 21.3%) under normoxic conditions (Figure 5D). In scratch assay, to make sure that the migration did not result from cell proliferation, the cells were serum‐starved to minimize proliferation for 24 h before the injury and treatment (Figure 5E). HUVECs were cultured in a six‐well plate and scratched using a pipette tip. After incubation for 24 h, the marked migration was observed in the scratch area, and the wound distance nearly disappeared after treatment with PFE‐1‐NPs@cp under light illumination. Over 70% wound healing area was obtained (Figure 5F). The difference in cell status between the control group and the other three groups after 24 h of hypoxic treatment may be attributed to the prolonged exposure to hypoxia, resulting in impaired cellular energy metabolism and limited proliferation rate. These results strongly proved the ability of PFE‐1‐NPs@cp to effectively relieve O_2_, indicating that PFE‐1‐NPs@cp can be used as an efficient O_2_ nanogenerator to protect against hypoxia and promote HUVECs migration.

### 2.4 Preparation and Characterization of Injectable and Light‐Triggered Hydrogel

**Figure 6:**
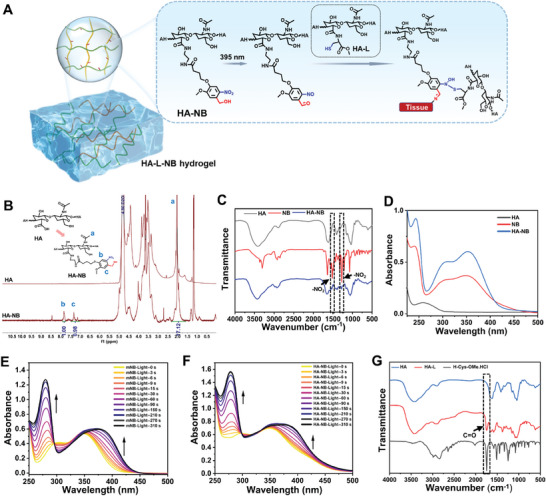
Synthesis and exploration of the crossing mechanism of HA‐L‐NB hydrogel. A) Schematic diagram of the strategies for light‐triggered gelation. B) Structure characterization of HA‐NB by ^1^H NMR. C) FTIR spectra of HA, NB, and HA‐NB. D) Absorption spectra of HA, NB, and HA‐NB. E) Absorption spectra of mNB with increasing irradiation time. F) Absorption spectra of HA‐NB with increasing irradiation time. G) FTIR spectra of HA, H‐Cys‐OMe HCl and HA‐L.

To deliver the PFE‐1‐NPs@cp to diabetic wounds and firmly retain them in the tissue, we synthesized a non‐radical‐polymerized hydrogel HA‐L‐NB. The gelation and tissue integration mechanism for HA‐L‐NB were shown in **Figure** **6**A. After exposure to illumination, the o‐nitrobenzyl carbinol labeled on the HA chain is converted into o‐nitrosobenzaldehyde by photochemical reaction. Then, the generated nitroso group is coupled with the thiol group on the other HA branch by a rapid SNO coupling reaction, resulting in the gelation of the hydrogel. Simultaneously, photogenerated aldehyde groups are anchored to the amino groups on the tissue, enabling in situ adhesion. The synthetic route of HA‐L‐NB was shown in Figure S2 (Supporting Information). First, mNB (a derivative of o‐nitrobenzyl carbinol) was synthesized according to the previous report.^[^ ^42^ ^]^ Then the photosensitive branch chain NB was further synthesized and characterized by FTIR spectrum, ^1^H NMR spectrum, and MALDI‐Mass spectrum (Figures S5‐S8, Supporting Information). Then the NB was linked to HA through amide linkage to form the biomacromolecule HA‐NB. As shown in Figure 6B, the proton on the N‐acetyl methyl group of HA still appeared at 1.90 ppm, and new peaks appeared at 7.90 and 7.30 ppm that corresponded to the protons on the phenyl group, which indicated the successful synthesis of HA‐NB. The ratio of the integrated area at 7.90 to the area at 1.90 ppm was calculated to be 17.5%, meaning a substitution degree of 17.5%. In addition, both HA‐NB and NB showed the stretching vibration peaks of ‐NO_2_ at 1527 and 1324 cm^−1^ in the infrared spectra (Figure 6C). The UV spectra showed that the maximum absorption peak of NB was at 350 nm, and that of HA‐NB was also at 350 nm (Figure 6D). These results further proved that the macromolecules HA‐NB were successfully prepared. In order to investigate the photoreaction HA‐NB, the control mNB was monitored by UV–vis absorption spectroscopy first. Upon 395 nm LED irradiation (10 mW cm^−2^), the maximum absorption of the mNB at 348 nm red‐shifted to 377 nm (Figure 6E), indicating the o‐nitrobenzyl carbinol transformed into o‐nitrosobenzaldehyde groups. In addition, the appearance of a peak related to the stretching vibration of C═O at 1696 cm^−1^ of the aldehyde group, and the disappearance of ‐NO_2_ stretching vibration at 1527 and 1324 cm^−1^ further confirmed the group transformation upon photoirradiation (Figure S9, Supporting Information). Interestingly, the maximum absorption of HA‐NB at 350 nm was also red‐shifted to 375 nm, which was similar to that of mNB, indicating the successful group transformation by photoreaction (Figure 6F). However, after irradiation at 395 nm, no obvious aldehyde proton was detected in HA‐NB, which could potentially be attributed to the labile nature of the protons on the aldehyde groups, resulting in their nondetection (Figure S10, Supporting Information). Finally, cysteine residues (from H‐Cys‐OMe) were grafted onto the backbone of another HA biomacromolecule, forming HA‐L. As can be seen from Figure S11 (Supporting Information), the proton on the N‐acetyl methyl group of HA still appeared at 1.90 ppm, along with new peaks corresponding to the proton of the cysteine residue. As shown in FTIR spectra (Figure 6G), the stretching vibration of C═O at 1740 cm^−1^ of the ester group of HA‐L indicated the synthesis of HA‐L.

**Figure 7:**
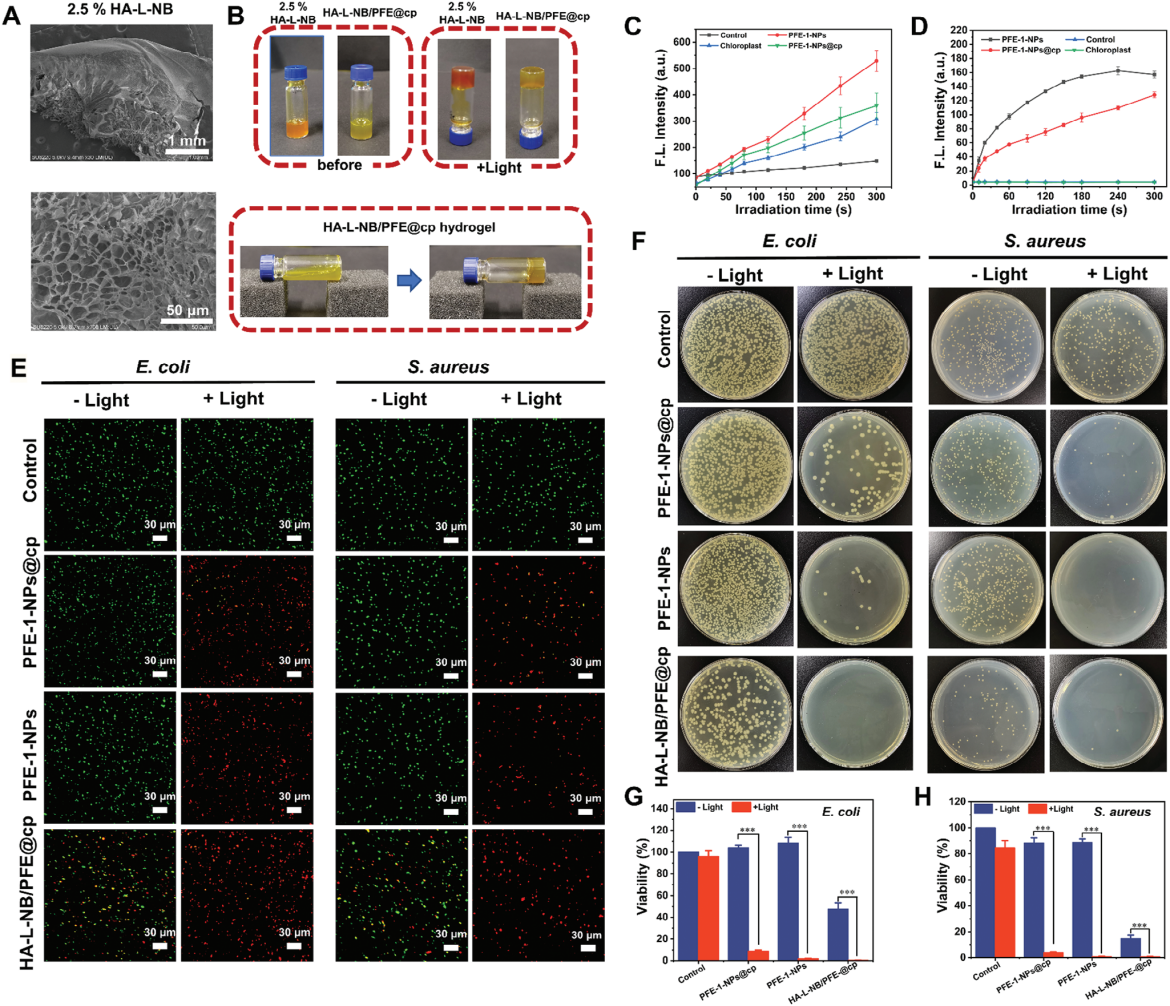
Gelation and antibacterial performance of HA‐L‐NB/PFE@cp hydrogel. A) SEM images of side view and cross‐section of HA‐L‐NB hydrogel. B) Injectability and gelation of HA‐L‐NB and HA‐L‐NB/PFE@cp hydrogel. C) ROS generation and D) ^1^O_2_ production under 395 nm light irradiation. Antibacterial performance: E) Bacterial live/dead staining of *S. aureus* and *E. coli* after treatments. Scale bar = 30 µm. F) Images of CFU assay. G, H) Quantitative analysis of CFU assay. The concentrations of PFE‐1‐NPs and chloroplasts were 6 µm and 5 µg mL^−1^, respectively. The mass fraction of HA‐L‐NB is 2.5%. The results represent mean ± standard deviation (*n* = 3). ^*^ *p* < 0.05, ^**^ *p* < 0.01, ^***^ *p* < 0.001 (Student's *t*‐test).

To visually observe the gelation of HA‐L‐NB, D‐PBS buffer solution (without calcium and magnesium) was used to prepare HA‐L‐NB pregel solution with different mass fractions (0.15, 0.30, 0.60, 1.25, and 2.50 wt.%). As shown in Figure S12 (Supporting Information), the HA‐L‐NB pregel solution began to gel at 0.30 wt.% mass fraction. Obviously, the HA‐L‐NB pregel solution was completely, stably, and fast‐gelled at 2.50 wt.%. Since the average pore size of a biological scaffold is a critical parameter affecting cell viability, attachment, and differentiation. The pore structure of HA‐L‐NB hydrogel was characterized by SEM. **Figure** **7**A showed that the hydrogel was highly porous with a moderately thick pore wall. The pore sizes ranged from 12.50 to 40 µm. Moreover, the mixtures of HA‐L‐NB pregel solution and PFE‐1‐NPs@cp were also able to remain injectable without illumination and quickly cross‐link under light irradiation (4 mW cm^−2^, 10 min) (Figure 7B). In order to evaluate the tissue binding strength of the hydrogels, HA‐L‐NB, and HA‐L‐NB/PFE@cp pregel solutions were used to perform in situ gelation experiments on pork surfaces (Figure S13, Supporting Information). No matter the pork was stretched or twisted, HA‐L‐NB and HA‐L‐NB/PFE@cp hydrogel on the surface showed no fracture and detachment, indicating that HA‐L‐NB/PFE@cp hydrogel had quite strong tissue binding strength. The results suggested that HA‐L‐NB can be applied as a carrier to deliver PFE‐1‐NPs@cp to diabetic wounds and can stably remain in the tissue. Additionally, to study the diffusion of growth factors in the gel, the diffusion behavior of the species in the hydrogel was simulated by selecting a nerve growth factor (NGF). The HA‐L‐NB hydrogel was homogeneously dispersed in deionized water to ensure adequate swelling of the hydrogel, and 100 µg mL^−1^ of NGF was added into the hydrogel samples to make NGF fully interact with the gel. The diffusion coefficient (D) of NGF was 2.34 × 10^−9^ cm^2^ s^−1^ and the coherence length was 18.3 nm by DLS. ^[^ ^43^ ^]^ The swelling properties of HA‐L‐NB were further characterized by weight ratio ^[^ ^44^ ^]^ and it was found that the swelling equilibrium could be reached within 30 min (swelling ratio of 175.6% ± 5.4%, Figure S14, Supporting Information). Then the rheological behaviors of the HA‐L‐NB hydrogel were evaluated with a rheometer in strain sweep mode. As shown in Figure S15A (Supporting Information), the hydrogel exhibited a linear viscoelastic region from 0.1% to 56% at a constant frequency of 1 rad s^−1^. At the studied frequency range, 0.1–100 rad s^−1^, the storage modulus (G′) was higher than the loss modulus (G″), being elastic dominant, which was a typical property of solid‐like hydrogels (Figure S15B, Supporting Information). The hydrogel exhibited both elastic and viscous characteristics, demonstrating the ability to conform to different deep and irregular wound shapes. To study the effect of different hydrogel extracts on cell proliferation, in vitro cytotoxicity of hydrogel extracts to HUVECs was measured. After incubation of cells with different extracts (HA‐NB, HA‐L‐NB, HA‐L‐NB/PFE@cp) for 24 h, cell viability still exceeded 100% (Figure S16, Supporting Information). After incubation for 48 h, the cell viability of HA‐L‐NB/PFE@cp + Light group reached 148%± 3.5% under light treatment, indicating that HA‐L‐NB/PFE@cp hydrogel had excellent biocompatibility and was able to promote cell proliferation.

### 2.5 Evaluation of Antibacterial Performance

The hyperglycemic environment in the wound of diabetic patients provides favorable conditions for bacterial colonization and growth, which can easily lead to multiple mixed bacterial infections. Therefore, the antibacterial capability is key for a hydrogel to improve wound healing. The production of ROS, including ^1^O_2_ by PFE‐1‐NPs@cp under light irradiation was then investigated to ensure photodynamic sterilization. With increasing irradiation time, the emission intensity of singlet oxygen sensor green (SOSG, ^1^O_2_ probe) and 2′,7′‐dichlorodihydrofluorescein diacetate (DCFH‐DA, ROS probe) increased rapidly in both PFE‐1‐NPs group and PFE‐1‐NPs@cp group, which demonstrated that ^1^O_2_ and ROS were produced effectively by PFE‐1‐NPs and PFE‐1‐NPs@cp (Figure 7C,D), indicating a promising antibacterial photodynamic therapy (aPDT) ability.

Then, the antibacterial activity of the hydrogels against model bacteria, *Escherichia coli* (*E. coli* ) and *Staphylococcus aureus* (*S. aureus*), was evaluated through bacterial live/dead staining by SYTO and propidium iodide (PI) staining. All live bacteria were stained by SYTO and emitted green fluorescence, whereas the dead bacteria were labeled by PI and emitted red fluorescence. Figure 7E showed that both *S. aureus* and *E. coli* in the control groups and the nonlight groups (PFE‐1‐NPs and PFE‐1‐NPs@cp) presented noticeable green fluorescence, and only a few bacteria were labeled with red fluorescence. In contrast, some bacteria appeared red fluorescence in the HA‐L‐NB/PFE@cp group under dark likely due to the antibacterial activity of HA.^[^ ^45^ ^]^ Furthermore, the strongest red fluorescence occurred in HA‐L‐NB/PFE@cp hydrogel groups under 395 nm light irradiation, indicating the highly effective antibacterial activity by combining aPDT of PFE‐1‐NPs with HA. Moreover, the colony‐forming unit (CFU) was evaluated to explore the antibacterial ability of the HA‐L‐NB/PFE@cp hydrogel. As shown in Figure 7F, compared with the nonlight groups, few *E. coli* and *S. aureus* colonies were observed in the PFE‐1‐NPs@cp, PFE‐1‐NPs, and HA‐L‐NB/PFE@cp groups under light excitation. The quantitative calculation (Figure 7G,H) showed after exposure to light, the survival rate of *E. coli* and *S. aureus* in the PFE‐1‐NPs group was 1.9% and 0.8%, respectively. And the survival rates of *E. coli* in the PFE‐1‐NPs@cp and HA‐L‐NB/PFE@cp groups were 8.8% and 0.3%, and that of *S. aureus* were 4.1% and 0.7%, respectively. Similarly, SEM images revealed significant damage to the integrity of the bacterial membrane in the PFE‐1‐NPs@cp + Light and PFE‐1‐NPs + Light groups in both bacteria. In contrast, most of the bacteria in the control and no‐light groups remained viable and structurally intact (Figure S17, Supporting Information). These results showed that PFE‐1‐NPs, PFE‐1‐NPs@cp, and HA‐L‐NB/PFE@cp had significant aPDT after light treatment. Importantly, HA‐L‐NB/PFE@cp hydrogel had the best antibacterial activity due to aPDT and HA dual biocidal performance, which should be more effective in preventing further wound infection.

### 2.6 In Vivo Wound Healing Enhanced by HA‐L‐NB/PFE@cp Hydrogel

**Figure 8:**
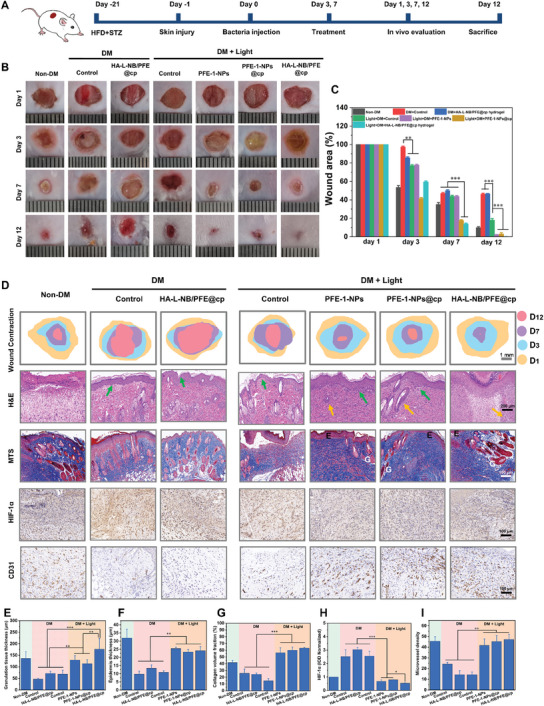
The in vivo therapeutic effect of HA‐L‐NB/PFE@cp on infected diabetic wounds. A) Schematic depiction of the treatment regime. B) Photographs of the infected wounds exposed to different treatments on days 1, 3, 7, and 12. C) Wound area analysis after various treatments. D) Schematic images of wound contraction from day 1 to day 12 and Hematoxylin and eosin (H&E) staining (The green arrows indicate the re‐epithelialization area, the yellow arrows indicate newly formed dermal), Masson's trichome staining (E: epidermis, G: granulation tissue), IHC of HIF‐1α and CD31 in each group at the end of treatment. Relative quantitative analysis of E) granulation tissue thickness, F) epidermis thickness, and G) collagen volume fraction. H) The HIF‐1α‐positive cell density and I) the CD31‐positive microvessel density at the end of treatment. The concentrations of PFE‐1‐NPs and chloroplasts were 6 µm and 5 µg mL^−1^, respectively. The mass fraction of HA‐L‐NB is 2.5%. The results represent mean ± standard deviation (*n* = 3). ^*^ *p* < 0.05, ^**^ *p* < 0.01, ^***^ *p* < 0.001 (Student's *t*‐test).

Due to the global increase in the prevalence of DM, improving delayed wound healing of infectious diabetes is of great significance in this field. To realize wound healing, it is required to simultaneously resolve these problems, such as infection, uncontrolled inflammation, and compromised angiogenesis, which is unanimously considered difficult.^[^ ^11^, ^46^ ^]^ To verify whether HA‐L‐NB/PFE@cp can promote wound healing of infectious diabetic wounds under light irradiation, a mouse model of infected diabetic chronic wounds was constructed and evaluated as described in the experimental section (**Figure** **8**A). The diabetic mice were divided into non‐diabetic groups and diabetic groups, and diabetic groups included non‐light group (PBS, HA‐L‐NB/PFE@cp) and light group (PBS, PFE‐1‐NPs, PFE‐1‐NPs@cp, HA‐L‐NB/PFE@cp). After sterilization of the back, a full‐thickness skin wound of 6 mm in diameter was created on the dorsal side. The infected wound model was established by instilling 10 µL of *S. aureus* suspension (3 × 10^7^ CFU mL^−1^) into the wound. Figure 8B showed representative images of the infected wounds in different groups from days 1 to 12. The wound healing effect in group “DM+Light, PFE‐1‐NPs” was much better than group “DM+Light, control” at days 7 and 12. This was mainly due to the promising antibacterial photodynamic therapy ability of PFE‐1‐NPs under light excitation. Interestingly, compared with untreated diabetic mouse wounds, the wound healing rate of HA‐L‐NB/PFE@cp + Light group was significantly faster. Especially on day 7, the wound had formed scab tissue. Until day 12, the wounds of this group had fully recovered and presented a smooth surface, whereas wounds were still visible in the other groups. The wound area during the treatment was analyzed according to the wound images (Figure 8C). The wound area of HA‐L‐NB/PFE@cp + Light group was 13.8%± 1.0% on Day 7. On day 12, the wound area of the DM + Control group was still 46.0% ± 1.0%. However, that of HA‐L‐NB/PFE@cp + Light group was close to zero. Additionally, the wound healing was significantly faster in the light groups compared to the nonlight groups. These results suggested that HA‐L‐NB/PFE@cp hydrogel can significantly promote wound healing in diabetic mice.

Wound healing is a well‐orchestrated biological process that involves anti‐inflammation, cell proliferation and migration, angiogenesis, epithelialization, and extracellular matrix deposition.^[^ ^46^, ^47^, ^48^, ^49^ ^]^ To verify the above healing results, the detail of the wound healing was assessed by the histological changes in skin tissues. Wound tissue for healing and angiogenesis was assayed by hematoxylin and eosin (H&E) staining, Masson's trichrome staining, and immunohistochemistry (IHC) on day 12 (Figure 8D). The statistical measurements of granulation tissue length showed that the wound contraction was faster in the HA‐L‐NB/PFE@cp + Light group than in the other groups. Dense granulation tissue and reepithelialization were observed on day 12 (Figure 8E,F). The granulation tissue thickness of the “DM+Light, HA‐L‐NB/PFE@cp” group was significantly different from that of “DM, HA‐L‐NB/PFE@cp” group (^***^ *p* < 0.001), and in the “DM+Light ”group, HA‐L‐NB/PFE@cp was significantly different from the other two groups (^**^ *p* < 0.01). Additionally, wounds treated with HA‐L‐NB/PFE@cp + Light generated significantly more collagen (blue stained) than those treated with other dressings (Figure 8G). The above results indicated that HA‐L‐NB/PFE@cp hydrogel promoted the granulation, reepithelialization, and collagen generation of refractory nonhealing wounds in diabetes.

HA‐L‐NB/PFE@cp + Light group can attenuate the hypoxia of diabetic wounds. To illustrate whether the rapid wound closure of the HA‐L‐NB/PFE@cp + Light group might be attributed to its delivery of oxygen to the wound tissue, alleviating hypoxia. Additional IHC analysis with HIF‐1α antibody (DAB coloration) was performed. The results showed that the density of HIF‐1α positive cells in the HA‐L‐NB/PFE@cp + Light group was significantly lower than that in other groups (Figure 8H), confirming that this hydrogel can continuously ameliorate hypoxic conditions in diabetic wounds. In the proliferation phase, the occurrence of neovascularization is critical to ensure the transport of nutrients and oxygen to the wound site to sustain fibroblast proliferation, collagen synthesis, and reepithelialization. Thus, immunostaining of the platelet endothelial cell adhesion molecule­1 (CD31) was performed to assess the presence of newly generated vessels in the granulation tissue on day 12. More CD31‐positive microvessels were observed in the light group than nonlight group. Especially, the HA‐L‐NB/PFE@cp + Light group showed the highest CD31‐positive microvessels density (47.33 ± 4.72) (Figure 8I). The biological safety of HA‐L‐NB/PFE@cp hydrogel was evaluated by taking samples from the main organs (heart, liver, spleen, lung, and kidney) of mice in each group and performing H&E staining (Figure S18, Supporting Information). The results showed that the treatment of samples did not cause substantial lesions in the main organs of mice, indicating that HA‐L‐NB/PFE@cp hydrogel had good biological safety. Altogether, our study showed that HA‐L‐NB/PFE@cp + Light can ameliorate hypoxic conditions, and further facilitate refractory wounds healing in diabetes.

## 3 Conclusion

In summary, a light‐triggered hydrogel oxygen self‐supply system was designed and developed for chronic wound healing in diabetes. The great spectral overlap between conjugated polymer emission and photosynthetic organisms accelerated the electron transport rate of photosystem II (PS II), thus augmenting photosynthesis beyond natural chloroplasts. Light‐triggered hydrogels were anchored on wound tissue in situ under light irradiation. Photosynthesis‐enhanced oxygen release promoted cell migration. Additionally, the biocompatible system effectively killed bacteria through photodynamic therapy, preventing further infection of the wound. Both in vivo and in vitro results demonstrated that the oxygen self‐supply system can simultaneously relieve wound hypoxia, eliminate bacteria, and promote cell migration, leading to the acceleration of wound healing. Overall, this study provides a promising approach for diabetic wound therapy. Furthermore, the proposed strategies are also potential for the treatment of other complex diseases, such as delayed fracture healing or cancer therapy.

## Conflict of Interest

The authors declare no conflict of interest.

## Author Contributions

Y.Q. conducted the synthesis, characterization, cell studies, animal experiments, and data analysis. Y.Q., Y.J., and L.L. performed the construction of a mouse model of infected diabetic chronic wounds. Y.Q. and T.Y. wrote the manuscript. Y.Q., B.B., Z.X., and L.M. contributed to the data analysis and discussed the data. T.Y. and Y.Q. conceived and designed the experiments. Y.T. supervised the study.

## Supporting information

Supporting Information

## Data Availability

The data that support the findings of this study are available from the corresponding author upon reasonable request.
