# Supplementary material for: Conjugated Polymer Composite Nanoparticles Augmenting Photosynthesis‐Based Light‐Triggered Hydrogel Promotes Chronic Wound Healing
Source: Adv Sci (Weinh). 2023 Nov 29;11(3):2304048. doi: 10.1002/advs.202304048 (PMC10797435; doi:10.1002/advs.202304048)
Supplement: Supplementary file 1 — Supporting Information [file ADVS-11-2304048-s001.pdf]

ADVANCED SCIENCE
Open Access

Supporting Information

for *Adv. Sci.*, DOI 10.1002/advs.202304048

Conjugated Polymer Composite Nanoparticles Augmenting Photosynthesis-Based Light-Triggered Hydrogel Promotes Chronic Wound Healing

*Qiong Yuan, Jia Yin, Ling Li, Benkai Bao, Xinyi Zhang, Meiqi Li and Yanli Tang**

Supporting Information

Conjugated Polymer Composite Nanoparticles Augmenting Photosynthesis-Based Light-Triggered Hydrogel Promotes Chronic Wound Healing

*Qiong Yuan, Jia Yin, Ling Li, Benkai Bao, Xinyi Zhang, Meiqi Li, Yanli Tang**

Key Laboratory of Applied Surface and Colloid Chemistry, Ministry of Education, Key Laboratory of Analytical Chemistry for Life Science of Shaanxi Province, School of Chemistry and Chemical Engineering, Shaanxi Normal University, Xi'an 710119, P. R. China

E-mail: yltang@snnu.edu.cn

Experimental Section S-3
Supporting Figures S-17
Figure S1. .... S-3
Figure S2. .... S-11
Figure S3. .... S-17
Figure S4. .... S-17
Figure S5. .... S-18
Figure S6. .... S-18
Figure S7. .... S-19
Figure S8. .... S-19
Figure S9. .... S-20
Figure S10. .... S-20
Figure S11. .... S-21
Figure S12. .... S-21
Figure S13. .... S-22
Figure S14. .... S-22
Figure S15. .... S-23
Figure S16. .... S-23
Figure S17. .... S-24
Figure S18. .... S-24
Figure S19. .... S-25
Figure S20. .... S-25
References S-26

Experimental Section

1. Synthesis and characterization of PFE-1-NPs@cp

1.1 Synthesis of conjugated polymer PFE-1

PFE-1 was synthesized by five steps from 2, 7-dibromofluorene according to the literature. [1]

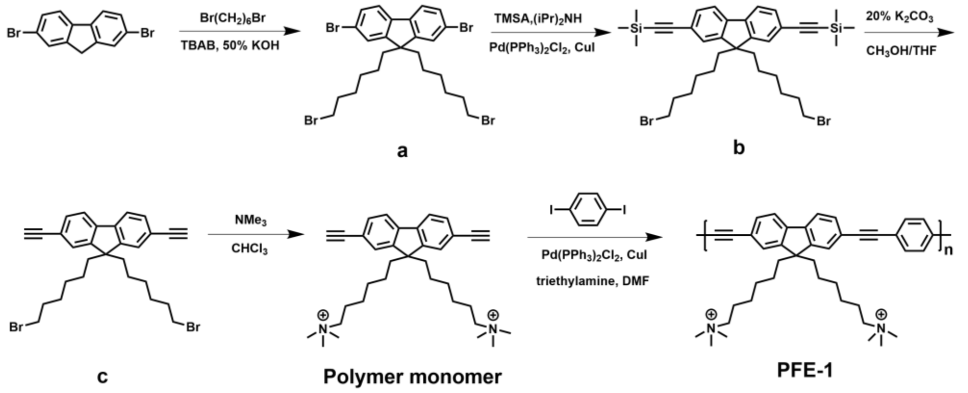

Figure S1. The synthesis of conjugated polymer PFE-1.

Firstly, 1, 6-dibromohexane was introduced to the side chain of 2, 7-dibromofluorene and salted to provide hydrophilicity. Then the alkyne group was introduced via trimethylsilylacetylene (TMSA) and the monomer was obtained by removing trimethylsilyl under alkaline conditions. Finally, the conjugated polymer PFE-1 was obtained by Sonogashira coupling reaction with Pd/Cu catalyst. ^{1}H NMR (400 MHz, DMSO) δ 7.96 - 7.38 (m,10 H), 3.42 (br, 4 H), 2.98(s, 18 H), 2.08 (br, 4 H),1.22 - 0.50 (m,16 H).

1.2 Chloroplast isolation and chlorophyll determination

Intact chloroplasts were isolated from commercially available fresh spinach. All preparations must be operated at 0 - 4 °C in the dark. The spinach was placed in advance in the fridge overnight to reduce the starch content. Fresh and thick spinach leaves were

selected and cut into as small pieces as possible, then soaked in precooled extraction buffer (0.4 mol/L sucrose, 0.05 mol/L Tris-HCl pH 7.6, 0.01 mol/L NaCl). The homogenate was homogenized at low speed for 10 seconds, repeated 3 times, filtered with 2 layers of filter paper and centrifuged at 200 g for 5 min, and the supernatant was centrifuged at 1100 g for 15 min to obtain the crude chloroplast extract. In order to further improve the integrity of chloroplasts, chloroplasts were purified by the double gradient method. Specifically, the crude chloroplasts extract was carefully and gently spread between the density gradient light solution and centrifuged at 3200 g for 15 min at 4 °C. After centrifugation, three layers of green bands could be seen in the centrifuge tube. The green layer between 40% density gradient light liquid and 80% density gradient heavy liquid was intact chloroplasts. Mixing 4.9 mL of acetone and 0.1 mL of chloroplast suspension intensively followed by removing the debris by centrifugation for 5 min at 3000 rpm, the chlorophyll acetone solution was obtained, and then the chlorophyll concentration was determined by measuring its absorbance at 652 nm in acetone.[2]

$$C\ (\mathrm{mg\ mL^{-1}}) = (OD_{652}\ \mathrm{nm} \times 100) / 36$$

1.3 Preparation of PFE-1-NPs

5 mL of PFE-1 tetrahydrofuran solution was prepared, and quickly added to 15 mL deionized water under the condition of ice bath ultrasound, then ultrasonic treatment for 5 min. THF was removed by injecting nitrogen into the mixture, and the solvent was concentrated to 5 mL by heating to 90 °C, and then filtered through a 0.22 μm filter to obtain nanoparticles. Hydrated particle size of the nanoparticles was measured by

Laser particle size analyzer (NanoBrook 90 PlusPALS).

1.4 Characterization of PFE-1-NPs@cp

At 4 °C, 6 μM PFE-1-NPs and 5 μg/mL chloroplast suspension were mixed and shaken for 6 h. Free nanoparticles were removed by centrifuging the suspension (3000 rpm, 3 min). The precipitate was then collected and suspended in sucrose buffer (pH 7.3). 0.5% glutaraldehyde was fixed overnight and washed twice with sterile water. Then ethanol (20, 40, 50, 70, 90 and 100%, respectively) was gradually added to the samples. Finally, the ethanol was replaced with tert-butanol: ethanol = 1:1, pure tert-butanol in turn, and 10 μL of suspension drops of PFE-1-NPs@cp complex was added dropwise to lyophilize the samples on silicon wafers. Samples were photogra phed and observed by SEM (SU8220, Hitachi, Tokyo, Japan) after 30 s (30 mA) of gold spraying by ion sputtering.

1.5 Energy transfer characterization

We hypothesized that chloroplasts as energy receptors, and the concentration of chloroplasts should be adjusted to observe the fluorescence spectral changes. The concentration of PFE-NPs was fixed at 1 μM, the chloroplast concentrations were 0.5 μg/mL to 30 μg/mL, and the PFE-NPs and chloroplast suspensions were mixed and incubated at 4 °C. The fluorescence spectra were measured at the excitation wavelength of 393 nm by fluorospectro photometer (F-7000, Hitachi, Tokyo, Japan).

1.6 Photosynthetic activity of PFE-1-NPs@cp

The photosynthetic activity of the PFE-1-NPs@cp complex was revealed by the Hill reaction. As an artificial electron acceptor, DCPIP (2, 6-dichlorophenol indophenol),

can capture electrons transferred from PS II to PS I in the photoreaction of chloroplasts. Therefore, the DCPIP reduction assay is a common method for estimating PS II activity. Brifly, chloroplast suspension (5 μg/mL), DCPIP (60 μM) and PFE-1-NPs (0, 2, 6, 12 μM) were mixed and the absorbance of the solutions at 600 nm was measured after light irradiation times of 0, 1, 2, 3, 4 and 5 min by UV spectrophotometer (UV-2600i, Shimadzu, Kyoto, Japan), respectively. The light intensity of the irradiation was 1, 4 and 8 mW/cm^2.

1.7 Reactive oxygen generation

2',7'-Dichlorodihydrofluorescein diacetate (DCFH-DA) could enter into living cells and then be deacetylated by intracellular esterase to form DCFH, which could be further oxidized by ROS to generate fluorescent 2.7-dichlorofluorescein (DCF). Thus, the fluorescence intensity of DCF reflects the ROS level. 500 μL of 1 mM DCFH-DA was added to 2 mL of 0.01 N NaOH solution react for 30 min in the dark. 10 mL of 25 mM PBS (pH 7.4) was added to obtain 40 μM DCFH and stored on ice. PFE-1-NPs, chloroplasts, and PFE-1-NPs@cp were dissolved in 40 μM DCFH, respectively, and the final solution contained 6 μM PFE-1-NPs, 5 μg/mL chloroplast. Next, with 5 mins of UV irradiation (395 nm, 4 mW/cm^2), the emission intensity changes at 525 nm were recorded. The excitation wavelength was 488 nm.

The SOSG (Singlet oxygen sensor green) probe was used to detect production of singlet oxygen ($^1O_2$) induced by PFE-1-NPs@cp. Specifically, 100 μg of SOSG dye was dissolved in 33 μL of methanol to obtain 5 mM SOSG stock solution, which was diluted to 1 μM SOSG with Tris-HCl (100 mM, pH 7.5). 2 mL of the 1 μM SOSG

solution was added with PFE-1-NPs, chloroplast, and PFE-1-NPs@cp, respectively, and the final solution contained 6 μM PFE-1-NPs, 5 μg/mL chloroplast. Next, with 5 mins of UV irradiation (395 nm, 4 mW/cm^2), the emission intensity changes at 525 nm were recorded every 1 min and the excitation wavelength was 504 nm.

1.8 Oxygen generation in solution

Samples were added to the 48-well plate, containing $[Ru(dpp)_3]Cl_2$ (10 mg/mL, 10 μL) after incubation for 15 min, and additionally, D-PBS was used as a control group. A small animal fluorescence imaging system (Xtreme II, Bruker, German, λ_{ex} = 488 nm, λ_{em} = 620 nm) to record the fluorescence.

2 The effect of PFE-1-NPs@cp complex on angiogenesis in vitro and wound healing

2.1 Cell viability assay

The cytotoxicity of PFE-1-NPs and chloroplasts was investigated by MTT (3-(4, 5-dimethyl thiazole-2-l)-2, 5-diphenyltetrazolium bromide tetrazolium) colorimetric assay. Human umbilical vein endothelial cells HUVECs and fibroblast NIH3T3 were selected for the experiment. Cells in the exponential growth phase were seeded in 96-well plates at 7000/well. After 24 h of incubation, the medium was replaced with fresh medium containing a range of different concentrations of PFE-1-NPs (0, 1, 2, 4, 6, 8, 10, 15 μM), chloroplast suspension (0, 1, 2, 4, 6, 8, 10, 15 μg/mL) and continued to incubate for 48 h. 10 μL of working MTT solution (5 mg/mL) was added to each well and cultured for 4 h. Finally, MTT solution was replaced by DMSO to solubilize formazan crystals and absorbance was spectrophotometrically recorded at 490 nm on a

microplate reader (SpectraMax M5, USA).

2.2 Glucose intervention levels

Hypoxic conditions were produced using the BIONIX-3 hypoxic culture kit (Sugiyama-gen). To determine the appropriate glucose intervention levels, the different glucose concentrations of the complete medium were divided into the normoxia groups and hypoxia groups (24 h). The glucose concentrations were 25 mM, 30 mM, 40 mM, 50 mM, 60 mM. HUVECs were inoculated in 96-well plates at 5000 cells/well, the DMEM complete medium without serum and different glucose concentrations was added, and five replicate wells were set up for each group. The cells were incubated under hypoxic and normoxic conditions for 24 h. 10 μL of MTT solution (5 mg/mL) was added to each well. After 4 h, the medium was removed and 100 μL of DMSO was added to each well and shaken 10 min. The absorbance values (OD) of each well were measured at 490 nm using microplate reader.

2.3 Measurement of intracellular oxygen content

The production of O_2 by chloroplasts photosynthesis in HUVECs was investigated by O_2 probe $[(Ru(DPP)_3)]Cl_2$. The fluorescence of $[(Ru(DPP)_3)]Cl_2$ can be strongly quenched by O_2. HUVECs were seeded and cultured for 24 h in the lower chamber of 24-well transwell inserts with 0.4-μm pore-sized filters at 37 °C. The medium contain PFE-1-NPs@cp was added in upper chamber and illuminated at 395 nm, 4 mW/cm^2 for 30 min. Then the 24-well transwell inserts were incubated under normoxic or hypoxic conditions for 24 h. After that, the cells were washed with PBS. $[(Ru(DPP)_3)]Cl_2$ probe (5 μM) was added and incubated for 30 min, PBS was washed twice, and then

Hochest33342 probe (10 μg/mL) was added and incubated for 10 min. Then the cells were washed with PBS and the medium was replaced with phenol red-free DMEM. The photographic observation was carried out by laser scanning confocal microscope (FV1200, OLYMPUS, Japan) and the average fluorescence intensity of the pictures was quantified by Image J software. $[(Ru(DPP)_3)]Cl_2$ probe, λ_{ex} = 488 nm, λ_{em} = 580-630 nm; Hochest 33342 probe, λ_{ex} = 405 nm, λ_{em} = 425-475 nm.

2.4 Scratch wound-healing assay

HUVECs at logarithmic growth stage were inoculated at 2×10^5 cells/well in a 6-well plate (n = 4 for each group). The monolayer was then scraped with a 200 μL pipette tip, washed, and optical images of the 0 h scoring site were taken with an optical microscope. Next, the medium was replaced with serum free high glucose medium containing PFE-1-NPs@cp treated with light at 4 mW/cm^2 for 30 min. The cells were rapidly incubated in hypoxia for 24 h and optical images were taken using an optical microscope. The distances between two sides of the scratch were measured using Image J software.

2.5 Transwell migration assay

Cell migration assay were performed by using 24-well transwell inserts with 8-μm pore-sized filters. Briefly, HUVECs were resuspended in high sugar medium and inoculated at 1×10^5 cells/well into the upper chamber. the lower chamber was added with PFE-1-NPs@cp with and without light treatment. After 24 h of anoxic incubation, the chamber was removed and the medium was aspirated and placed in a new 24-well plate. Cells were fixed with 4% paraformaldehyde (20 min at 37 °C) and permeabilized

with 100% methanol for 20 min. The migrated cells kept on the upper surface filter were removed by cotton swab, while the migrated cells on the bottom side of the filter were stained with a 0.1% crystal violet solution for 15 min. Migration of HUVECs was evaluated by viewing under high-power magnification and counting the migrated cells in five randomly selected fields/well viewed.

2.6 Tube-formation assay

The tube formation assay was performed by using 24-well transwell inserts with 0.4 μm pore-sized filters. 250 μL per well of thawed matrigel was added into the lower chamber of a pre-cooled 24-well plate and incubated for 1 h at 37°C. HUVECs were then seeded into the lower chamber of the 24-well plate (5×10^4 cells/well). Then PFE-1-NPs@cp was added to the lower chamber. After light treatment, cells were incubated in anoxic environment for 3 h, normoxia was restored for 3 h. To assess the tube formation, HUVECs was stained with Calcein-AM and imaged under an inverted fluorescence microscope (IX73, OLYMPUS, Japan) and quantified using Image J software.

3. Synthesis of HA-NB and HA-L:

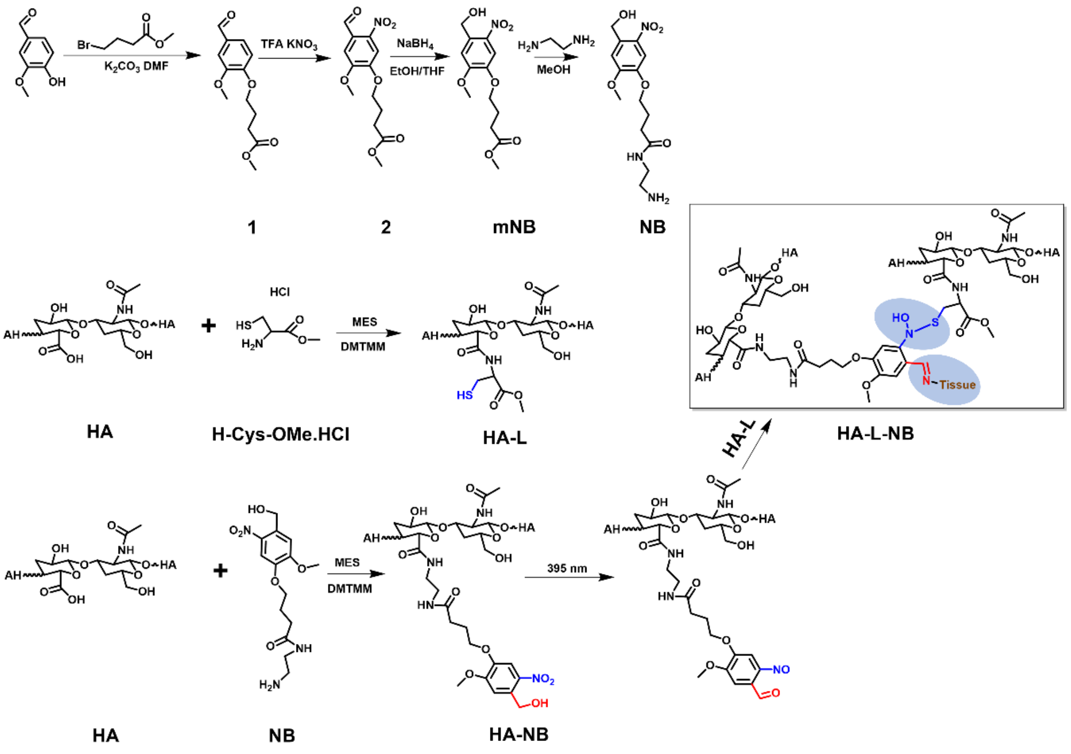

Figure S2. Structure and synthetic protocol for preparation of mNB, NB, HA-L, HA-NB and HA-L-NB.

3.1 Synthetic protocols

3.1.1 Synthesis of NB

NB was synthesized according to a previous reference (Figure S2, Figure S19, Figure S20).[3-4]

3.1.2 Synthesis of HA-L

L-cysteine methyl ester hydrochloride (50 mg, 0.29 mmol) was added to hyaluronic acid sodium (116.37 mg, 0.29 mmol of disaccharide unit, 70~2,000 kDa) in MES (0.01 M) solution (pH 5.3, 5 mL). Then, DMTMM (85 mg, 0.29 mmol) was dissolved in water (1.5 mL) and added into the above solution in three portions with 30 min intervals

between addition. The mixture was stirred for 4 h in the dark at 35 °C. Afterwards, 100% ethanol (80 mL) was added dropwise to the reaction mixture. The obtained yellow solid was redissolved in water (20 mL), and the mixture was treated with ethanol as described above. The crude product was frozen and lyophilized to give HA-L as a white powder (146 mg).

3.1.3 Synthesis of HA-NB

NB (14 mg, 0.042 mmol) was added to hyaluronic acid sodium (33.71 mg, 0.084 mmol of disaccharide unit, 70~2,000 kDa) in MES (0.01 M) solution (pH=5.3, 5 mL). Then, DMTMM (24.76 mg, 0.084 mmol) was dissolved in water (1.5 mL) and added into the above solution in three portions with 30 min intervals between additions, and the mixture was stirred for 4 h in the dark at 35 °C. Afterwards, 100% ethanol (80 mL) was added dropwise to the reaction mixture. The obtained yellow solid was redissolved in water (20 mL), and the mixture was treated with ethanol as described above. The crude product was frozen and lyophilized to give HA-NB as a yellow powder (40 mg).

3.2 Hydrogel preparation

HA-NB and HA-L samples were dissolved in Dulbecco's phosphate-buffered saline (D-PBS, pH 7.4, v/v=1:1) to obtain the HA-L-NB precursor solutions at different concentrations. Then PFE-1-NPs@cp was added to the HA-L-NB precursor solution to obtain the HA-L-NB/PFE@cp precursor solution. Next, the precursor solution was irradiated by a 395 nm LED UV irradiation (10 mW/cm^2) for 10 min to form the network of HA-L-NB/PFE@cp hydrogel. The network structure of the hydrogel was observed by scanning electron microscopy (SEM, SU8220, Hitachi, Tokyo, Japan)

3.3 Coherence length and diffusion coefficient

To detect the diffusion of growth factors such as vascular endothelial growth factor in the gel, the diffusion behavior of the species in the hydrogel was simulated by selecting a nerve growth factor (NGF). The HA-L-NB hydrogel was homogeneously dispersed in deionized water to ensure adequate swelling of the hydrogel, and 100 μg/mL of NGF was added into the hydrogel samples to make NGF fully interact with the gel. The autocorrelation function of the light scattering signal and the diffusion coefficient (D) of NGF were determined by dynamic light scattering (DLS).[5] The coherence length was further obtained by the following equation:

$$\text{Coherence length} = \sqrt{\frac{\tau \times D}{3}}$$

Where τ represents the autocorrelation time, which can be obtained from the fitted autocorrelation function. D is the diffusion coefficient.

3.4 Swelling analysis

To perform swelling analysis, 200 μL of hydrogel was weighed and recorded as W_0, then 2 mL of deionized water was added to swell, the water was blotted with filter paper at a preset time point, and the weight was recorded as W_d, the relative equilibrium swelling rate was calculated according to the following equation:

$$\text{Swelling ratio} = (Wd - W_0) / W_d$$

3.5 The rheological properties analysis

The rheological properties of the hydrogel were recorded with a rheometer (DHR-2, TA, USA), equipped with a parallel plate with the diameter of 20 mm. When tested, a part of the gel with the integrity was carefully taken. Strain sweeps were performed at

25 °C with a strain of 0.05-100 % and a frequency of 1 %. Oscillatory experiments were performed at 25 °C with a frequency of 0.1-100 rad $s^{-1}$and a strain of 1 %.

4. In vitro antibacterial assay

The antibacterial activity of HA-L-NB/PFE@cp hydrogel against *Staphylococcus aureus* (*S. aureus*) and *Escherichia coli* (*E. coli*) was investigated in vitro using the spread plate method. The bacterial concentration of different reagent mixtures (PBS, PFE-1-NPs, PFE-1-NPs@cp, HA-L-NB/PFE@cp hydrogel) was 1×10^7 CFU/mL. These samples were treated with or without irradiation (395 nm, 4 mW/cm^2, 30 min). Then, 100 μL of the diluted bacterial suspension was uniformly spread onto the fresh Luria Bertani broth agar plates. After cultivation 24 h, the bacterial colonies on the agar plates were photographed and counted. The bacteria suspension treated by PBS with or without irradiation was considered the control group.

In addition, we studied the antibacterial properties of hydrogels by a bacterial live/dead staining assay. Briefly, after different treatments, the bacteria cells were co-stained by PI and SYTO 9, PI and SYTO 24 for 20 min in the dark, followed by washing thrice with PBS. According to the manufacturer's instructions, all bacteria were labeled by SYTO9 and appeared green fluorescence, while dead bacteria were stained by PI and revealed red fluorescence. Finally, fluorescence images were captured using confocal laser scanning microscopy. (PI probe, λ_{ex} =559 nm, λ_{em} = 600-650 nm; Syto 24 or SYTO 9 probe, λ_{ex} = 488 nm, λ_{em} = 490-540 nm)

Besides, SEM imaging was also performed to visualize the bacteria morphological changes after the antibacterial experiments. Specifically, after treatments, all collected

bacteria were washed with PBS and then fixed with 2.5% glutaraldehyde solution at 4 °C for 40 min. After fixation, these specimens were serially dehydrated by graded ethanol solutions (20-100%). Finally, the ethanol was replaced with tert-butanol: ethanol = 1:1, pure tert-butanol in turn, and 10 μL of the sample was added dropwise to the silicon wafer for lyophilization. Next, the dried bacteria were sputter-coated with platinum to increase conductivity, and their morphologie were observed by SEM.

5. In vivo experiments in diabetic mice

The infected diabetic wound model was established to evaluate the therapeutic effect of the hydrogels. All animal operations and experimental procedures were approved by the Shaanxi Provincial Laboratory Animal Management Committee and the Ethics Committee of Shaanxi Normal University. Balb/C mice (male, about 25 g in weight) were obtained from the Shaanxi Normal University City Animal Experiment Center and housed in an SPF class laboratory animal room, fed with high-fat and high-sugar feed for 3 weeks (Shanghai Mao Kang Biotechnology Co., Ltd.). After 12 h of fasting, the animals other than those in the control group were administered STZ (100 mg/kg; Shanghai Mao Kang Biotechnology Co., Ltd.) mixed in sodium citrate buffer (intraperitoneal injection) daily for two consecutive days. All of the treated mice with plasma glucose levels ≥ 16.7 mM under fasting condition were considered diabetic. Mice were randomly divided into 7 groups (n = 4): (1) Healthy mice without any treatment;(2) Diabetic mice with PBS treatment; (3) Diabetic mice with HA-L-NB/PFE@cp treatment; (4) Diabetic mice with PBS + Light treatment; (5) Diabetic mice with PFE-1-NPs + Light treatment; (6) Diabetic mice with PFE-1-NPs@cp +

Light treatment; (7) Diabetic mice with HA-L-NB/PFE@cp + Light treatment. After mice were anesthetized with isoflurane (2%), the back was shaved, followed by creation of full-thickness ~ 6 mm diameter skin wound under sterile condition. Subsequently, 10 μL of PBS with *S. aureus* suspensions (3×10^7CFU/mL) was inoculated onto this wound. Then, wounds of all infected mice in different groups were treated with the above samples, respectively. After the predetermined post-treatment days (3, 7, 12 days), the skin wounds in different groups were photographed and measured. On the 3rd and 12th day, the skin tissues around the wound were harvested, and fixed with 4% paraformaldehyde buffer for H&E, Masoon and immunohistochemical (IHC) staining. Primary antibodies used in this research included HIF-1α (#41005, SAB, 1:100) and CD31 (ab182981, Abcam, 1:2000). Quantitative analysis of each sample was assessed with Image-Pro Plus 6.0 software.

6. Statistical analysis

All experiments were conducted at least three times unless otherwise noted and All results were shown as the mean ± standard deviation (SD). The statistical analysis was performed using Origin 2021, followed by a student's t-test and one-way analysis of variance. $^*P < 0.05$ was considered statistically significant. $^{**}P < 0.01$ and $^{***}P < 0.001$ were considered highly significant.

Supporting Figures

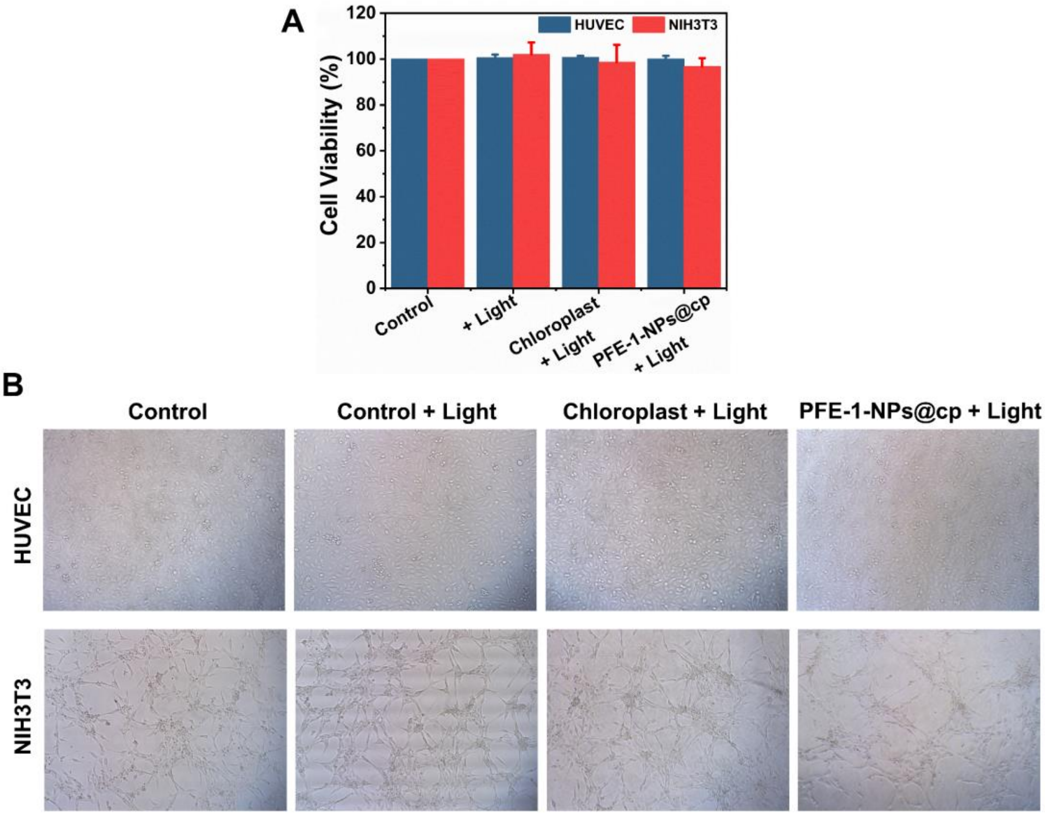

Figure S3. A) Cell viability of HUVECs and NIH3T3 cells incubated with chloroplast and PFE-1-NPs@cp after 395nm light illumination (4 mW/cm^2,30 min). B) The growth of the two kinds of cells under optical microscope.

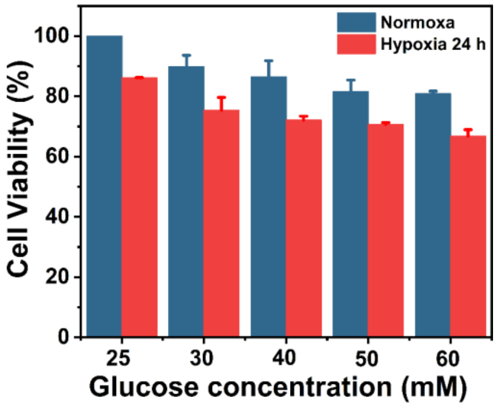

Figure S4. Cell viability of HUVECs incubated with different concentrations of glucose at normoxic and hypoxic condition.

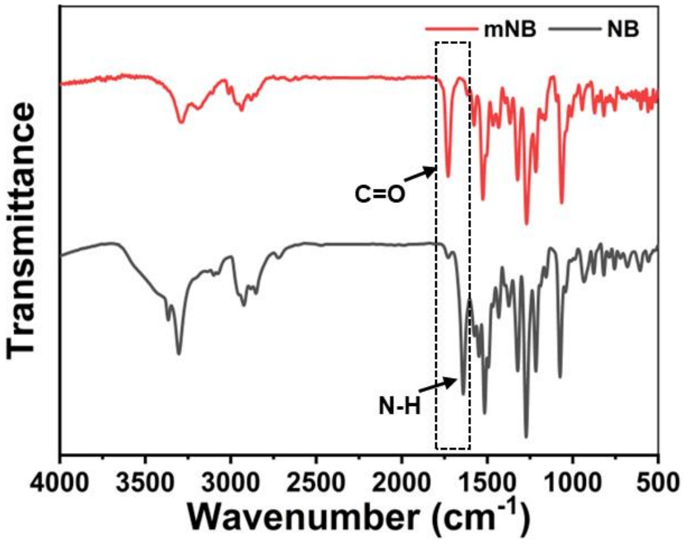

Figure S5. FTIR spectra of mNB and NB.

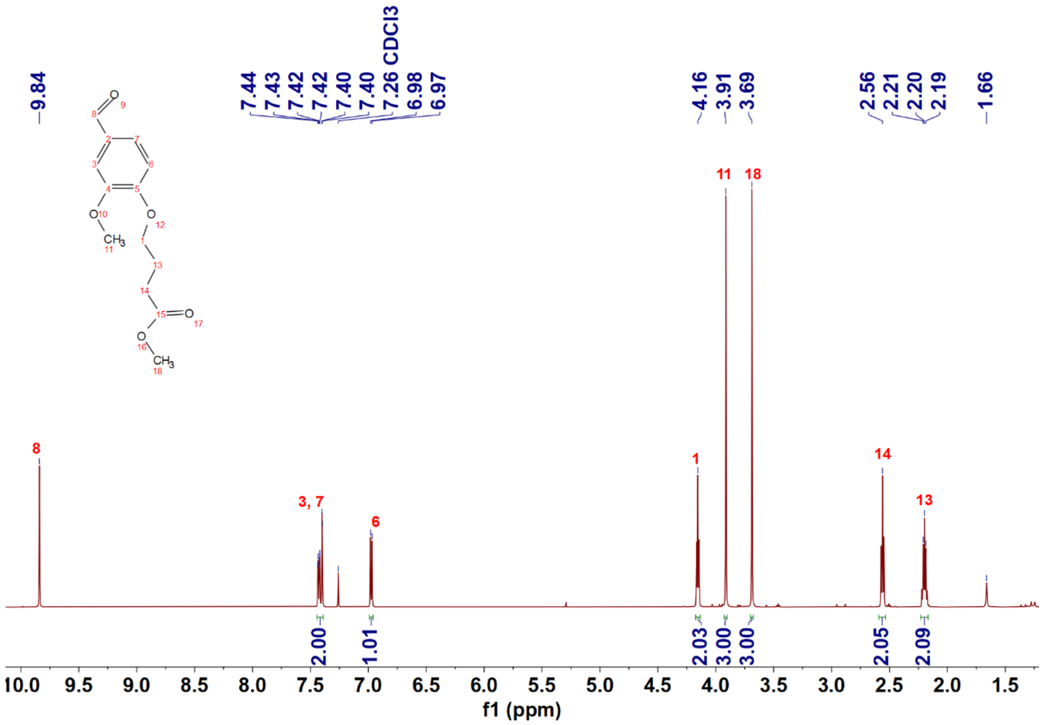

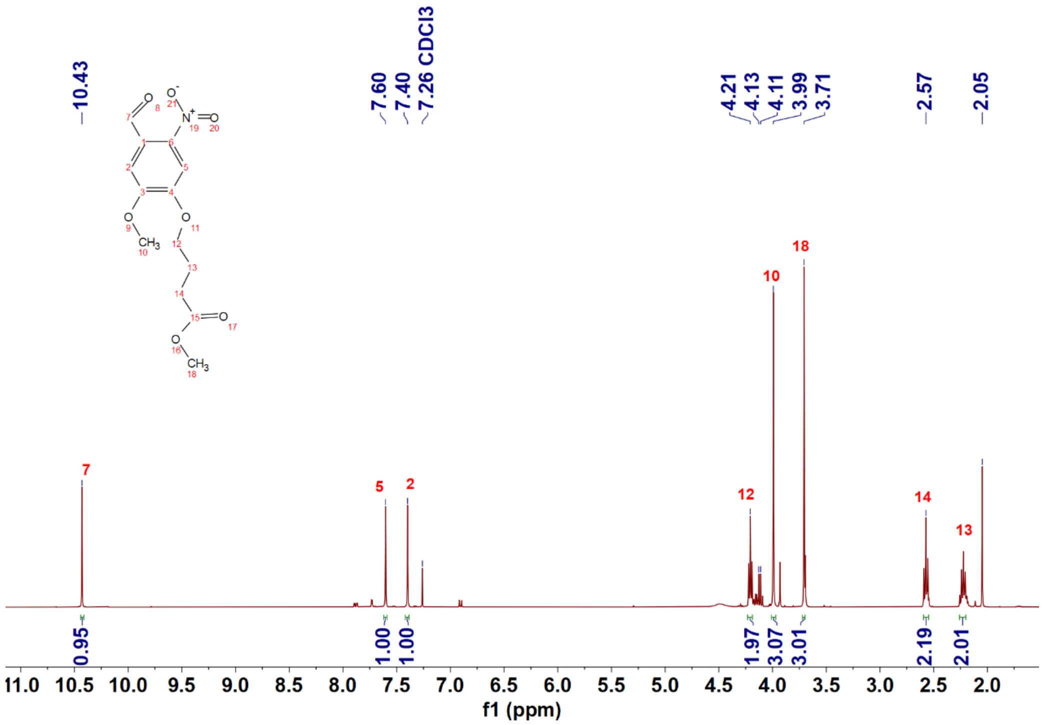

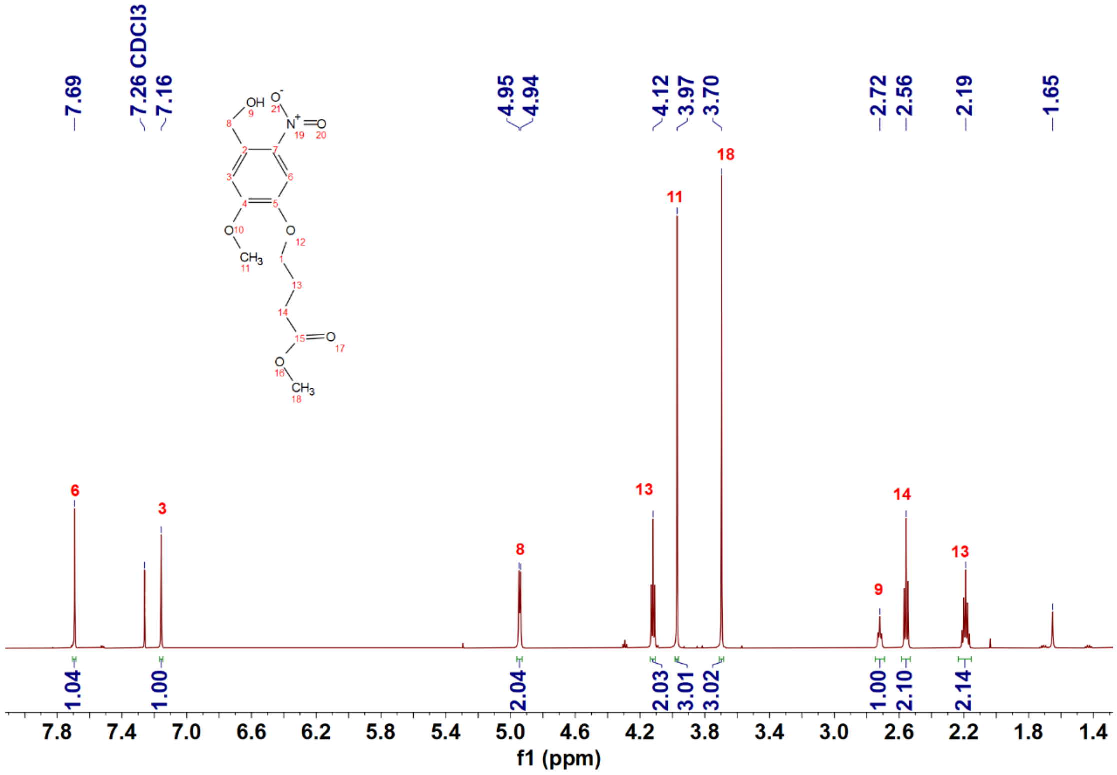

Figure S6. ^{1}H-NMR spectra (600 MHz) of compound 1, compound 2 and mNB in $CDCl_3$.

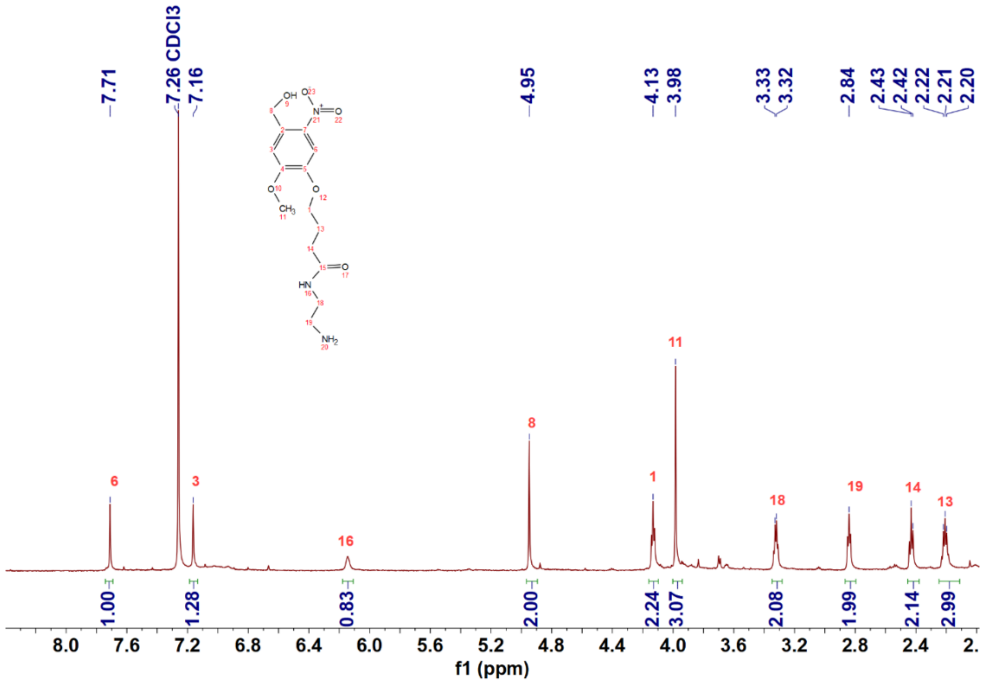

Figure S7. ^{1}H-NMR spectrum (600 MHz) of NB in $CDCl_3$.

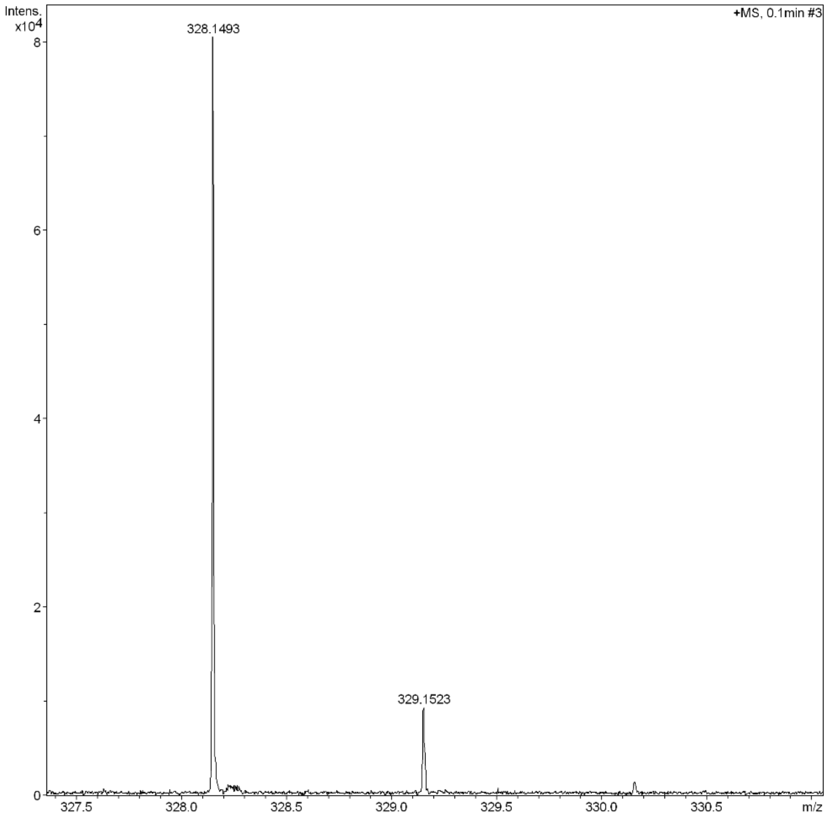

Figure S8. MALDI-Mass spectrum of NB (328.1493[M+H]$^{+}$).

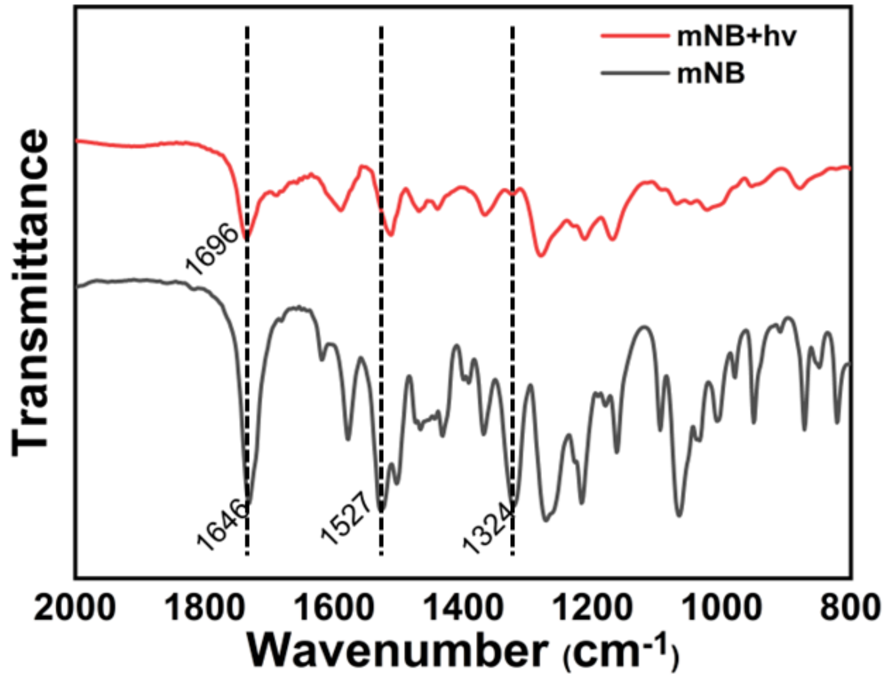

Figure S9. FTIR spectra of the mNB+hv and mNB.

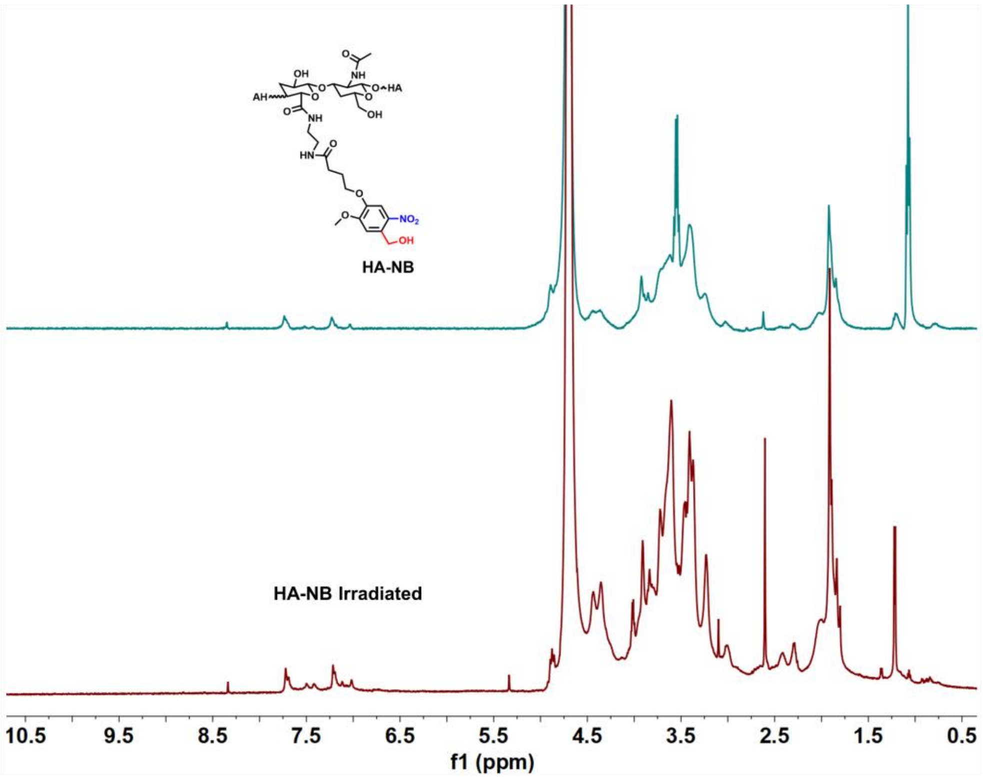

Figure S10. ^{1}H-NMR spectra (600 MHz) of HA-NB and HA-NB irradiated in D_2O.

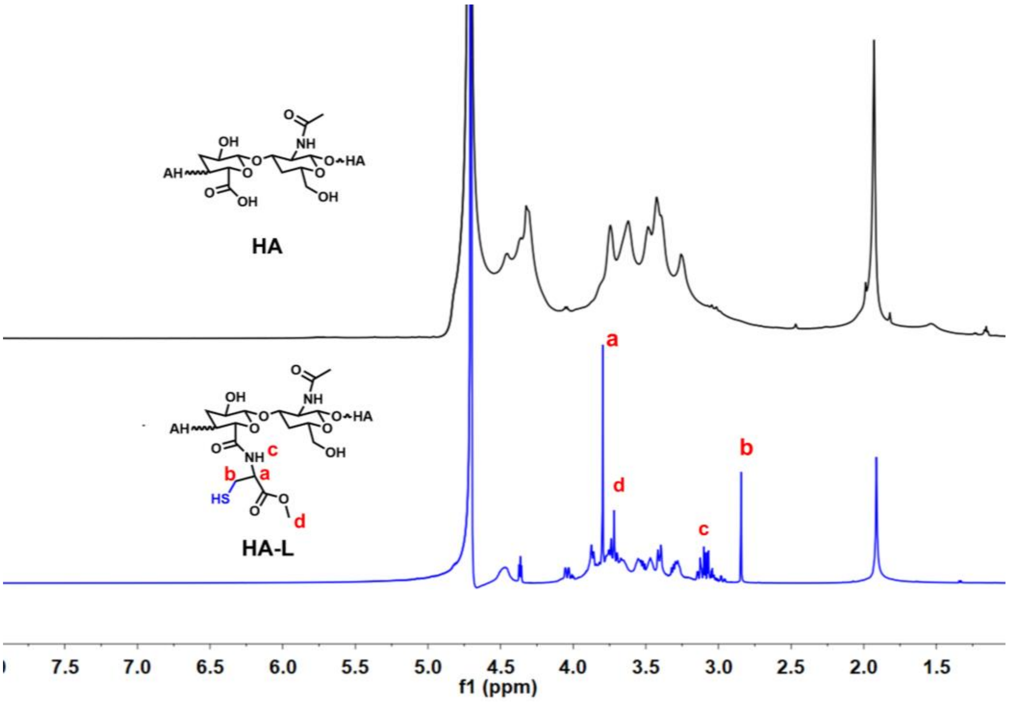

Figure S11. ^{1}H-NMR spectra (600 MHz) of HA and HA-L in D_2O.

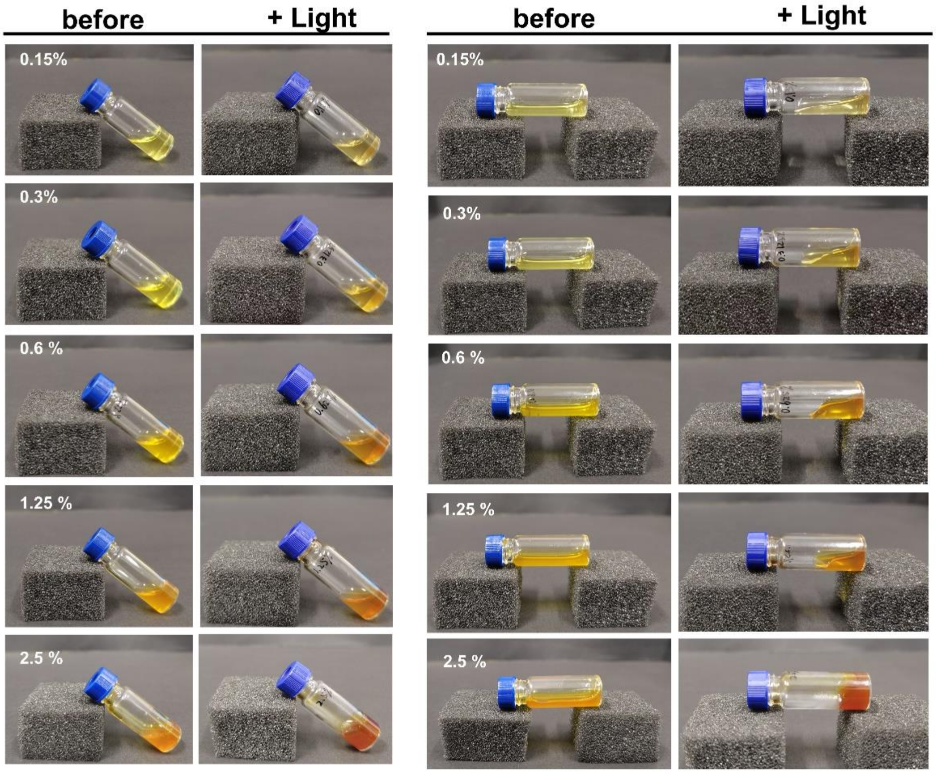

Figure S12. Macroscopic observation of 395 nm-induced HA-L-NB hydrogel formation. HA-L-NB hydrogel formation is observed at a minimum concentration of 2.5% in D-PBS.

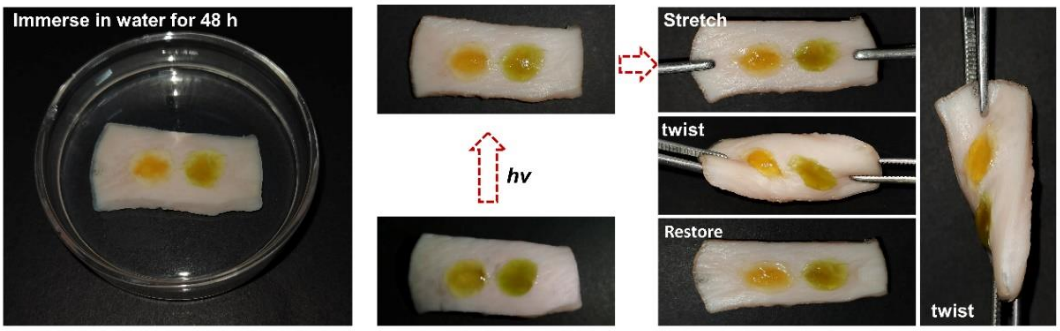

Figure S13. Photographs of the HA-L-NB and HA-L-NB/PFE@cp hydrogels in situ formingon pork tissue upon light irradiation. No breakage or detachment between the hydrogel and tissue is seen in the photographs. Stretch or twist, restore and immerse in water for 48 h.

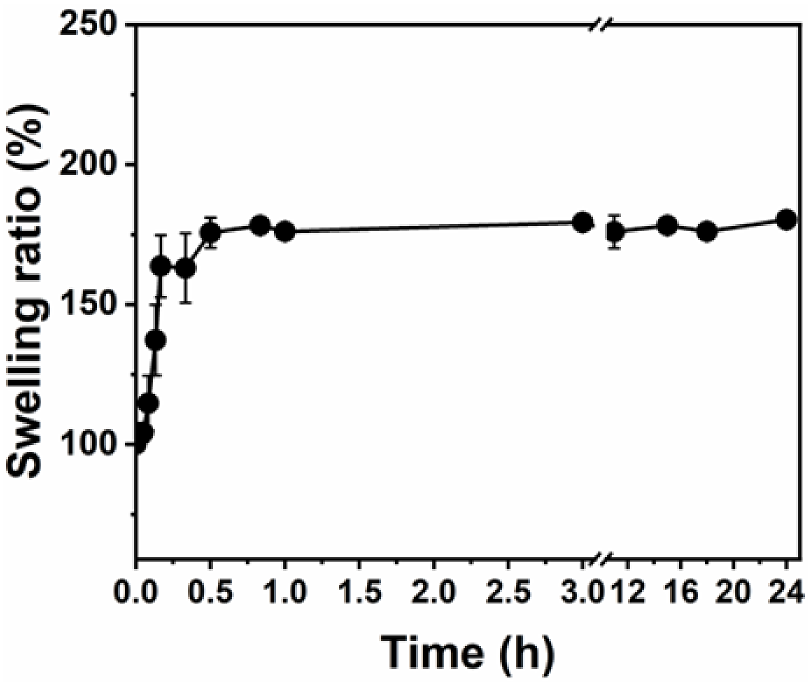

Figure S14. Swelling ratios of HA-L-NB hydrogel.

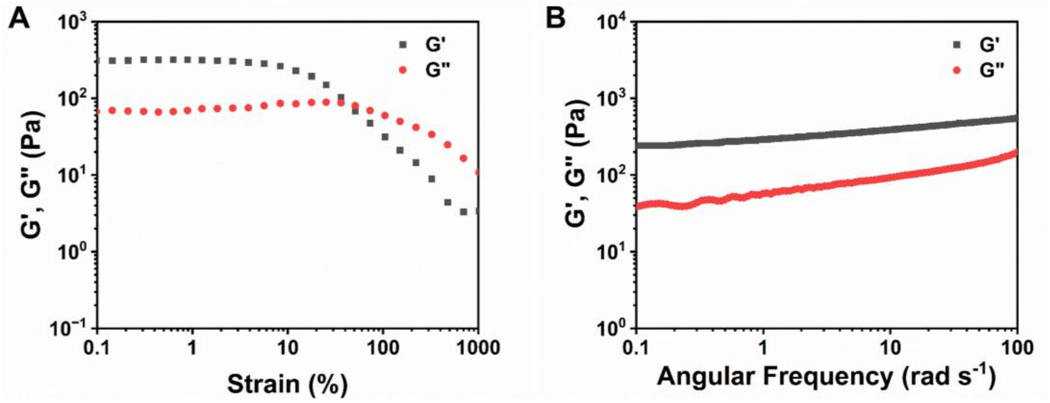

Figure S15. A) Strain dependent oscillatory rheology of the HA-L-NB hydrogel at frequency of 1 rad s^{-1}; B) Frequency sweep for HA-L-NB hydrogel with a frequency range of 0.1–100 rad s^{-1} at strain of 1%. T = 25.0 °C.

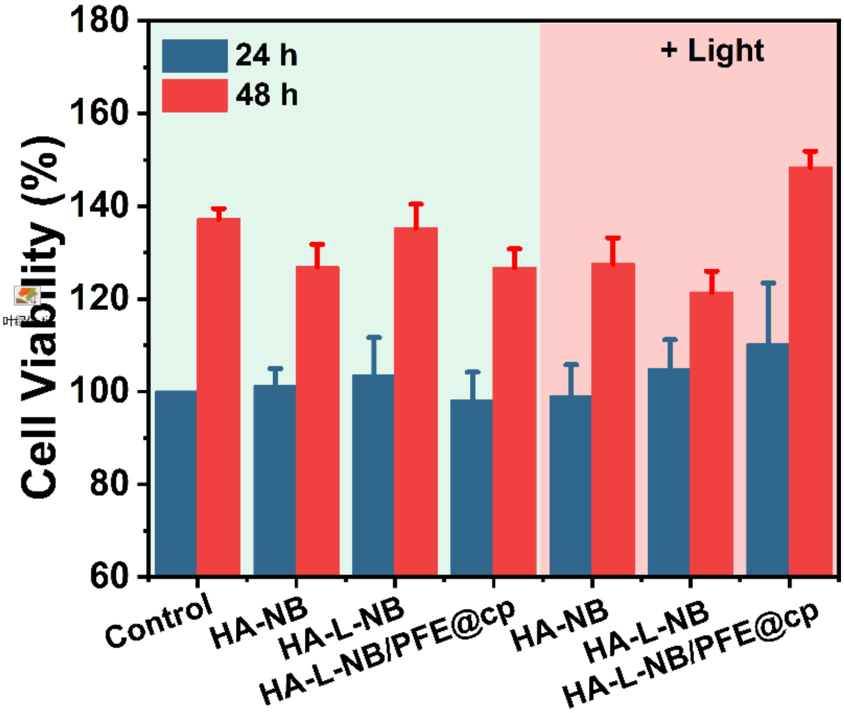

Figure S16. In vitro cytotoxicity to HUVECs after exposure to the extracts of hydrogel samples (HA-NB, HA-L-NB, HA-L-NB/PFE@cp) for 24 h and 48 h.

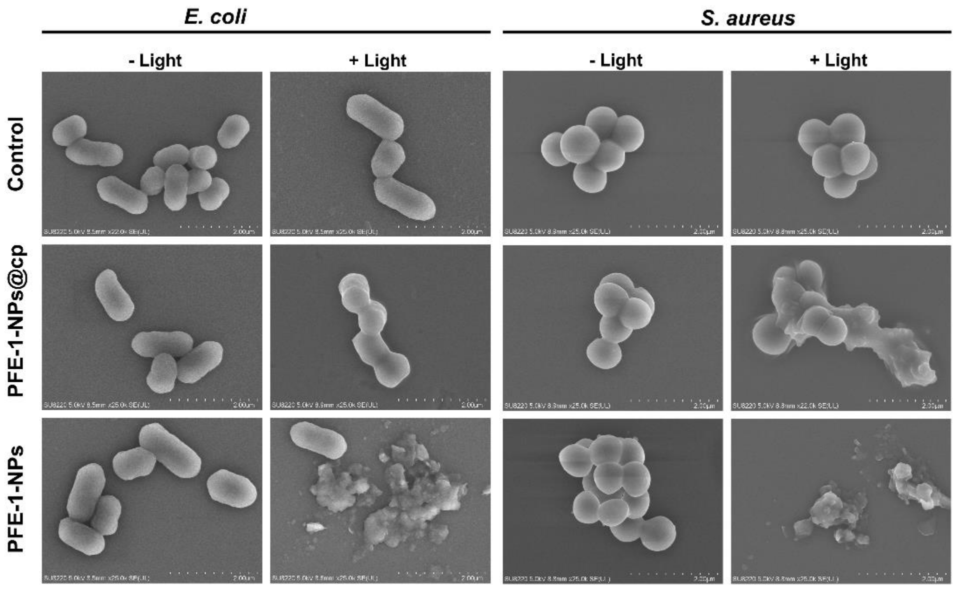

Figure S17. SEM photographs of *S. aureus* and *E. coli* after treatments with PBS, PFE-1-NPs@cp, PFE-1-NPs.

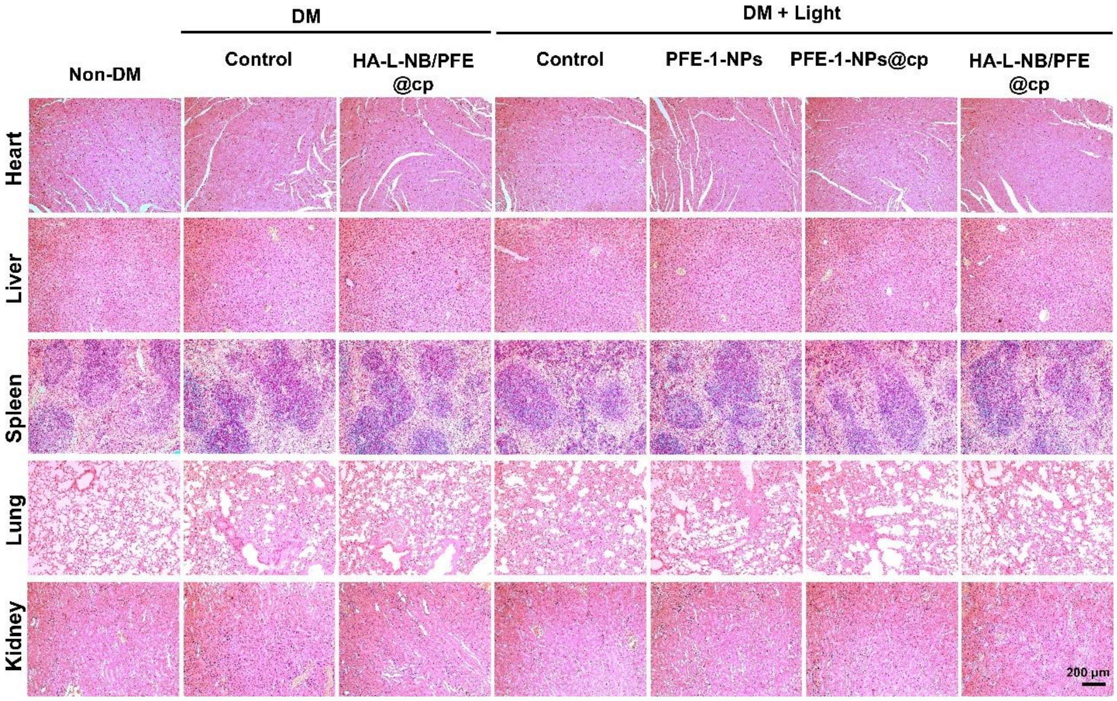

Figure S18. H&E staining of main organs (heart, liver, spleen, lung, and kidney) collected from rats in the different groups. Scale bar = 200 μm.

References

[1] L. Zhai, Z. Zhang, Y. Zhao, Y. Tang, *Macromolecules* **2018**, 51, 7239.

[2] X. Zhou, L. Zhou, P. Zhang, F. Lv, L. Liu, R. Qi, Y. Wang, M-Y. Shen, H-H. Yu, G. C. Bazan, S. Wang, *Adv. Electron. Mater.* **2019**, 5, 1800789.

[3] T. Pauloehrl, G. Delaittre, M. Bruns, M. Meissler, H. G. Borner, M. Bastmeyer, C. Barner-Kowollik, *Angew. Chem. Int. Ed.* **2012**, 51, 9181.

[4] Y. Yang, J. Zhang, Z. Liu, Q. Lin, X. Liu, C. Bao, Y. Wang, L. Zhu, *Adv. Mater.* **2016**, 28, 2724.

[5] S. M. Patil, D. A. Keire, K. Chen, *AAPS Adv. Pharm. Sci. Ser.* **2017**, 19, 1760.
